# Efficacy and Safety of Zilucoplan in Amyotrophic Lateral Sclerosis: A Randomized Clinical Trial

**DOI:** 10.1001/jamanetworkopen.2024.59058

**Published:** 2025-02-17

**Authors:** Sabrina Paganoni, Christina N. Fournier, Eric A. Macklin, Lori B. Chibnik, Melanie Quintana, Benjamin R. Saville, Michelle A. Detry, Matteo Vestrucci, Joe Marion, Anna McGlothlin, Senda Ajroud-Driss, Marianne Chase, Lindsay Pothier, Brittney A. Harkey, Hong Yu, Alexander V. Sherman, Jeremy M. Shefner, Meghan Hall, Gale Kittle, James D. Berry, Suma Babu, Jinsy Andrews, Derek Dagostino, Eric Tustison, Elisa Giacomelli, Erica Scirocco, Gustavo Alameda, Eduardo Locatelli, Doreen Ho, Adam Quick, Jonathan Katz, Daragh Heitzman, Stanley H. Appel, Sheetal Shroff, Kevin Felice, Nicholas J. Maragakis, Zachary Simmons, Timothy M. Miller, Nicholas Olney, Michael D. Weiss, Stephen A. Goutman, Joseph Americo Fernandes, Omar Jawdat, Margaret Ayo Owegi, Laura A. Foster, Tuan Vu, Hristelina Ilieva, Daniel S. Newman, Ximena Arcila-Londono, Carlayne E. Jackson, Shafeeq Ladha, Terry Heiman-Patterson, James B. Caress, Andrea Swenson, Amanda Peltier, Richard Lewis, Dominic Fee, Matthew Elliott, Richard Bedlack, Edward J. Kasarskis, Lauren Elman, Jeffrey Rosenfeld, David Walk, Courtney McIlduff, Paul Twydell, Eufrosina Young, Kristin Johnson, Kourosh Rezania, Namita A. Goyal, Jeffrey A. Cohen, Michael Benatar, Vovanti Jones, Jonathan Glass, Jaimin Shah, Said R. Beydoun, James P. Wymer, Lindsay Zilliox, Shakti Nayar, Gary L. Pattee, Jennifer Martinez-Thompson, Brittany Harvey, Shital Patel, Paul Mahoney, Petra W. Duda, Merit E. Cudkowicz

**Affiliations:** 1 Sean M. Healey & AMG Center for ALS and the Neurological Clinical Research Institute, Massachusetts General Hospital, Harvard Medical School, Boston; 2 Spaulding Rehabilitation Hospital, Harvard Medical School, Boston, Massachusetts; 3 Department of Neurology, Emory University, Atlanta, Georgia; 4 Biostatistics Center, Massachusetts General Hospital, Department of Medicine, Harvard Medical School, Boston; 5 Department of Epidemiology, Harvard T. H. Chan School of Public Health, Boston, Massachusetts; 6 Berry Consultants LLC, Austin, Texas; 7 Department of Neurology, Feinberg School of Medicine, Northwestern University, Chicago, Illinois; 8 Barrow Neurological Institute, Phoenix, Arizona; 9 Department of Neurology, Columbia University, New York, New York; 10 Phil Smith Neuroscience Institute, Holy Cross Hospital, Silver Spring, Maryland; 11 Department of Neurology, Nova Southeastern University, Fort Lauderdale, Florida; 12 Department of Neurology, Ohio State University, Columbus; 13 California Pacific Medical Center and Forbes Norris MDA-ALS Research and Treatment Center, San Francisco; 14 Texas Neurology, Dallas; 15 Methodist Neurological Institute, Houston, Texas; 16 Department of Neuromuscular Medicine, Hospital for Special Care, New Britain, Connecticut; 17 Department of Neurology, Johns Hopkins University School of Medicine, Baltimore, Maryland; 18 Department of Neurology, Penn State Milton S. Hershey Medical Center, Hershey, Pennsylvania; 19 Department of Neurology, Hope Center for Neurological Disorders, Washington University in St Louis, St Louis, Missouri; 20 Providence ALS Clinic, Portland, Oregon; 21 Department of Neurology, University of Washington Medical Center, Seattle; 22 Department of Neurology, University of Michigan, Ann Arbor; 23 Department of Neurology, University of Nebraska Medical Center, Omaha; 24 Departmennt of Neurology, University of Kansas Medical Center, Kansas City; 25 Department of Neurology, University of Massachusetts Medical School, Worcester; 26 Department of Neurology, University of Colorado School of Medicine, Aurora; 27 Department of Neurology, University of South Florida, Tampa; 28 Jefferson Weinberg ALS Center, Philadelphia, Pennsylvania; 29 Henry Ford Health System Department of Neurology, Detroit, Michigan; 30 Department of Neurology, University of Texas Health, San Antonio; 31 Department of Neurology, Temple Health, Philadelphia, Pennsylvania; 32 Department of Neurology, Wake Forest University School of Medicine, Winston-Salem, North Carolina; 33 Department of Neurology, University of Iowa, Iowa City; 34 Department of Neurology, Vanderbilt University Medical Center, Nashville, Tennessee; 35 Department of Neurology, Cedars-Sinai Medical Center, Los Angeles, California; 36 Department of Neurology, Medical College of Wisconsin, Milwaukee; 37 Department of Neurology, University of Virginia, Arlington; 38 Department of Neurology, Duke University, Durham, North Carolina; 39 Department of Neurology, University of Kentucky, Lexington; 40 Department of Neurology, University of Pennsylvania School of Medicine, Philadelphia; 41 Department of Neurology, Loma Linda University School of Medicine, Loma Linda, California; 42 Department of Neurology, University of Minnesota/Twin Cities ALS Research Consortium, Minneapolis and St Paul; 43 Department of Neurology, Beth Israel Deaconess Medical Center, Harvard Medical School, Boston, Massachusetts; 44 Department of Neurology, Spectrum Health Medical Group, Grand Rapids, Michigan; 45 Department of Neurology, SUNY (State University of New York) Upstate, Syracuse; 46 Department of Neurology, Ochsner Health System, New Orleans, Louisiana; 47 Department of Neurology, University of Chicago, Chicago, Illinois; 48 Department of Neurology, University of California, Irvine, Medical Center; 49 Department of Neurology, Dartmouth-Hitchcock Medical Center, Lebanon, New Hampshire; 50 Department of Neurology, University of Miami, Miami, Florida; 51 Department of Physical Medicine and Rehabilitation, School of Medicine, University of Missouri, Columbia; 52 Department of Neurology, Mayo Clinic, Jacksonville, Florida; 53 Department of Neurology, Keck School of Medicine, University of Southern California, Los Angeles; 54 Department of Neurology, University of Florida, Gainesville; 55 Department of Neurology, University of Maryland School of Medicine, Baltimore; 56 Department of Neurology, Georgetown University, Washington, DC; 57 Neurology Associates, Lincoln, Nebraska; 58 Department of Neurology, Mayo Clinic, Rochester, Minnesota; 59 UCB Pharma, Cambridge, Massachusetts; 60 UCB, Slough, United Kingdom

## Abstract

**Importance:**

The etiology of amyotrophic lateral sclerosis (ALS), a fatal neurodegenerative disease, is unknown. However, neuroinflammation and complement activation may play a role in disease progression.

**Objective:**

To determine the effects of zilucoplan, an inhibitor of complement C5, in individuals with ALS.

**Design, Setting, and Participants:**

Zilucoplan was tested as regimen A of the HEALEY ALS Platform Trial, a phase 2 to 3 multicenter, randomized, double-blind, placebo-controlled perpetual platform clinical trial with sharing of trial infrastructure and placebo data across multiple regimens. Regimen A was conducted from August 17, 2020, to May 4, 2022. A total of 162 participants were randomized to receive zilucoplan (122 [75.3%]) or regimen-specific placebo (40 [24.7%]). An additional 124 concurrently randomized participants were randomized to receive placebo in other regimens.

**Interventions:**

Eligible participants were randomized in a 3:1 ratio to receive zilucoplan or matching placebo within strata of edaravone and/or riluzole use for a planned duration of 24 weeks. Active drug (zilucoplan, 0.3 mg/kg) and placebo were provided for daily subcutaneous dosing.

**Main Outcomes and Measures:**

The primary end point was change in disease severity from baseline through 24 weeks as measured by the Amyotrophic Lateral Sclerosis Functional Rating Scale–Revised (ALSFRS-R) total score and survival, analyzed using a bayesian shared-parameter model and reported as disease rate ratio (DRR; <1 indicating treatment benefit). The study included prespecified rules for early stopping for futility. Outcome analyses were performed in the full analysis set comparing the zilucoplan group with the total shared placebo group (n = 164).

**Results:**

Among the 162 participants who were randomized (mean [SD] age, 59.6 [11.3]; 99 [61.1%] male), 115 (71.0%) completed the trial. The estimated DRR common to ALSFRS-R and survival was 1.08 (95% credible interval, 0.87-1.31; posterior probability of superiority, 0.24). The trial was stopped early for futility. No unexpected treatment-related risks were identified.

**Conclusions and Relevance:**

In this randomized clinical trial of zilucoplan in ALS, treatment did not alter disease progression. The adaptive platform design of the HEALEY ALS Platform Trial made it possible to test a new investigational product with efficient use of time and resources.

**Trial Registration:**

ClinicalTrials.gov Identifier: NCT04297683

## Key Points

**Question:**

What is the effect of zilucoplan, an inhibitor of complement C5, in amyotrophic lateral sclerosis (ALS)?

**Findings:**

This randomized clinical trial with 122 participants randomized to receive zilucoplan and 164 to receive placebo was stopped early for futility.

**Meaning:**

These results suggest that the use of zilucoplan, a complement inhibitor targeting C5, does not impact the progression of ALS.

## Teaser

This randomized clinical trial evaluates the safety, tolerability, and efficacy of zilucoplan in patients with amyotrophic lateral sclerosis.

## Introduction

Amyotrophic lateral sclerosis (ALS) is a fatal disease characterized by degeneration of motor neurons, leading to loss of muscle strength, impaired speech and swallowing, and weakness of the ventilatory muscles. Median survival is 2 to 3 years from onset, and current treatment options have only modest impacts on disease trajectory.

The etiology of ALS is unknown, but neuroinflammation is implicated as a possible driver of disease progression, and complement activation may play a role.^1^ Activation of the complement cascade has been demonstrated in biofluids and tissue samples from patients with ALS.^2,3,4^ Additionally, preclinical studies in ALS animal models have shown delayed onset of motor symptoms, improved motor function, and improved survival with C5-mediated complement inhibition.^5,6^

Zilucoplan is a small (15–amino acid), subcutaneously administered macrocyclic peptide inhibitor of complement C5 that has recently been approved for the treatment of acetylcholine receptor antibody–positive myasthenia gravis.^7^ It blocks cleavage of C5, activation of the terminal complement pathway, and assembly of the membrane attack complex, thereby preventing complement-mediated tissue damage and cell death.^8^ Herein we report the results of a randomized, placebo-controlled clinical trial of zilucoplan as tested in the HEALEY ALS Platform Trial,^9,10^ which evaluated the safety, tolerability, and efficacy of zilucoplan in individuals with ALS.

## Methods

### Trial Design and Oversight

Zilucoplan was evaluated in regimen A of the HEALEY ALS Platform Trial, a phases 2 to 3 randomized, double-blind, placebo-controlled clinical trial conducted at 54 Northeast ALS Consortium centers in the US between August 17, 2020, and May 4, 2022. The trial was conducted in accordance with Good Clinical Practice guidelines of the International Council for Harmonization and ethical principles of the Declaration of Helsinki.^11^ Protocol approval was provided for all trial sites by a single investigational review board, the Partners Human Research Committee. All participants were required to provide written informed consent prior to screening.

The trial was designed by and conducted through the Northeast ALS Consortium, a global collaborative trial network, in collaboration with UCB, Brussels, Belgium. Participant-level data were obtained at each trial site, then sent to the Coordination Center at Massachusetts General Hospital, Boston. An independent data and safety monitoring board reviewed unblinded safety data throughout the trial. Statistical analyses were performed by Berry Consultants and Massachusetts General Hospital Department of Neurology and Division of Biostatistics within the Department of Medicine. Trial reporting followed the Consolidated Standards of Reporting Trials (CONSORT) reporting guideline for randomized clinical trials.

### Trial Participants

Eligibility criteria for the HEALEY ALS Platform Trial included adults with a diagnosis of clinically possible, probable, laboratory-supported probable, or definite ALS defined by revised El Escorial criteria; disease duration of 36 months or less; a vital capacity of 50% or greater predicted for age, height, and sex; the ability to swallow pills and liquids; and either no use of riluzole and/or edaravone or stable dosing of riluzole for more than 30 days and/or 1 full treatment cycle of edaravone. Participants assigned to regimen A had to receive both quadrivalent and serotype B meningococcal vaccinations at least 14 days prior to the first dose of study drug, and participants were excluded from regimen A if they had a history of meningococcal disease or prior treatment with a complement inhibitor. Data on race and ethnicity were collected to help establish racial representation in this trial, explain trial generalizability to external populations, and encourage health equity following National Institute of Neurological Disorders and Stroke–recommended common data elements using fixed categories. Categories self-reported by participants included American Indian or Alaska Native, Asian, Black or African American, Native Hawaiian or Other Pacific Islander, White, or other (including multiracial, not reported, and unknown); ethnicity, as Hispanic or Latino or non-Hispanic or non-Latino. Full inclusion and exclusion criteria are provided in the study protocol (Supplement 1).

### Trial Interventions and Procedures

Eligible participants were randomized in a 3:1 ratio to receive zilucoplan or matching placebo within strata of edaravone and/or riluzole use for a planned duration of 24 weeks. Further information on randomization into regimens and within regimens is provided in the eMethods in Supplement 2. Active drug (zilucoplan, 0.3 mg/kg) and placebo were provided for subcutaneous administration for daily dosing. Clinic or phone visits were conducted at baseline and every 4 weeks thereafter through week 24, with a final phone follow-up at week 28. The full schedule of activities was outlined in the study protocol (Supplement 1). Participants who completed the randomized, double-blind clinical trial were eligible for enrollment in an open-label extension trial evaluating the long-term effects of zilucoplan.

### Outcomes

The primary efficacy outcome was change in the rate of disease progression, as measured by the Amyotrophic Lateral Sclerosis Functional Rating Scale–Revised (ALSFRS-R)^12^ and survival, from baseline through 24 weeks. Secondary clinical efficacy outcomes included the rate of decline in isometric muscle strength as measured by handheld dynamometry and the rate of decline in slow vital capacity. Additional prespecified outcomes included rates of decline in ALSFRS-R subdomains; quantitative voice characteristics as measured by a speech analytics platform (Speech Vitals; Aural Analytics); changes in biofluid markers of neurodegeneration and neuromuscular degeneration, including neurofilament light chain protein; change in respiratory function as assessed by home spirometry; and change in complement pathway biomarker levels in blood (as assessed by the Wieslab alternative complement pathway assay; Svar Life Science AB). Death or death-equivalent events (tracheostomy or permanent assisted ventilation >22 hours daily for more than 7 consecutive days),^13^ tracheostomy placement, and hospitalization events (excluding elective surgical procedures) were captured. A hierarchy was prepared for secondary outcomes for inference testing. Additional information about the outcomes of this study can be found in the eMethods in Supplement 2. The full statistical analysis plan is provided in Supplement 1.

### Sample Size Justification

A sample size of 160 patients per regimen with a randomization ratio of 3:1 active treatment to placebo and sharing of controls across at least 3 concurrently enrolling regimens provided approximately 80% power to detect a 30% slowing in disease progression (common across mortality and function) and a 1-sided type I error less than 2.5%. Pretrial simulations for power were previously described.^10^

### Analytic Populations

The primary analytic population, the full analysis set (FAS), included all participants randomized to the active treatment arm, all participants randomized to the placebo arm within the regimen (regimen-specific placebo), and participants randomized to placebo from other contributing regimens. Observations completed after regimen lock and participants determined to not meet ALS diagnostic criteria were excluded. Additional prespecified efficacy analyses were performed in the regimen-specific population, which limited placebo groups to those randomized within the zilucoplan regimen. The FAS included both the regimen-specific placebo group and placebo groups from other contributing regimens (shared placebo group). Sensitivity analyses that limit comparison with the regimen-specific placebo group are referred to as the efficacy regimen only. Safety analyses were performed in the FAS, including all participants who initiated treatment. Participants were analyzed according to the treatment group to which they were randomized within their respective regimen. Information on handling of intercurrent events and description of estimand are included in the eMethods in Supplement 2.

The FAS dataset included longitudinal data on disease progression (ALSFRS-R measurements at 0, 4, 8, 12, 16, 20, and 24 weeks; at early termination visits; and assessments during double-blind follow-up that substitute for missed scheduled visits) and data on survival during the planned 24 weeks of participation. Follow-up for primary analysis of survival among participants who did not die or reach a death equivalent prior to the week 24 visit was censored at the week 24 visit date, if completed, the date of consent withdrawal, if withdrawn, or the last date at which vital status is known prior to the end of the week 24 visit window for participants lost to follow-up. Off-schedule results (eg, those collected as part of an early termination visit) were used in place of the closest missing scheduled visit (while preserving visit sequence) for analyses that depend on visit-specific data. Off-schedule results that do not substitute for a missed schedule visit were not used.

### Statistical Analysis

The primary analysis was a bayesian shared parameter model of function and survival that provided an integrated estimate of the relative rate of disease progression on treatment relative to control (denoted as disease rate ratio [DRR]). A DRR less than 1 indicated a slowing in disease progression on treatment relative to control. Within the multicomponent model, ALSFRS-R was measured for those who survived using a linear repeated measures model that adjusts for baseline covariates and accommodates potential differences in the shared control across regimens, and survival was measured through an exponential proportional hazards model.^10^ Sensitivity analyses were performed to assess the slowing in disease progression separately across the 2 components to allow for an assessment of the contribution of the individual components. Additional information on the primary analysis methods, including interpretation of the estimand, are provided in the eMethods in Supplement 2.

Prespecified interim analyses were conducted within the master protocol with the potential for a regimen to stop early for futility. Interim analyses occurred simultaneously for all actively enrolling regimens with sufficient data. Interims began when at least 1 regimen within the master protocol had 40 randomized participants (30 to active treatment and 10 to control) with the opportunity to complete at least 24 weeks of follow-up. Subsequent interims occurred every 12 weeks.

Futility was to be declared for a regimen at an interim analysis if the regimen had the minimum required data (40 randomized participants with the opportunity to complete at least 24 weeks of follow-up) and the posterior probability that the treatment slowed disease progression by at least 10% was less than 5%. Stopping due to early success was not considered. In the event of futility, resources saved from avoiding the open-label extension stage of the trial were estimated by calculating the sum of all expected visits that would have occurred between the actual and projected last participant last visit (LPLV) and the number of doses that would have been administered through the projected completion of the full 24-week trial. Individual participants’ visit schedules and projected dates of trial completion were generated from the electronic data capture system. Secondary outcomes methods are provided in the eMethods in Supplement 2.

Statistical analyses were performed using SAS, version 9.4 (SAS Institute Inc); R, version 4.1.2 (R Project for Statistical Computing); and JAGS, version 4.2.0. Two-sided *P* < .05 indicated statistical significance.

## Results

### Trial Participants

**Figure 1.**
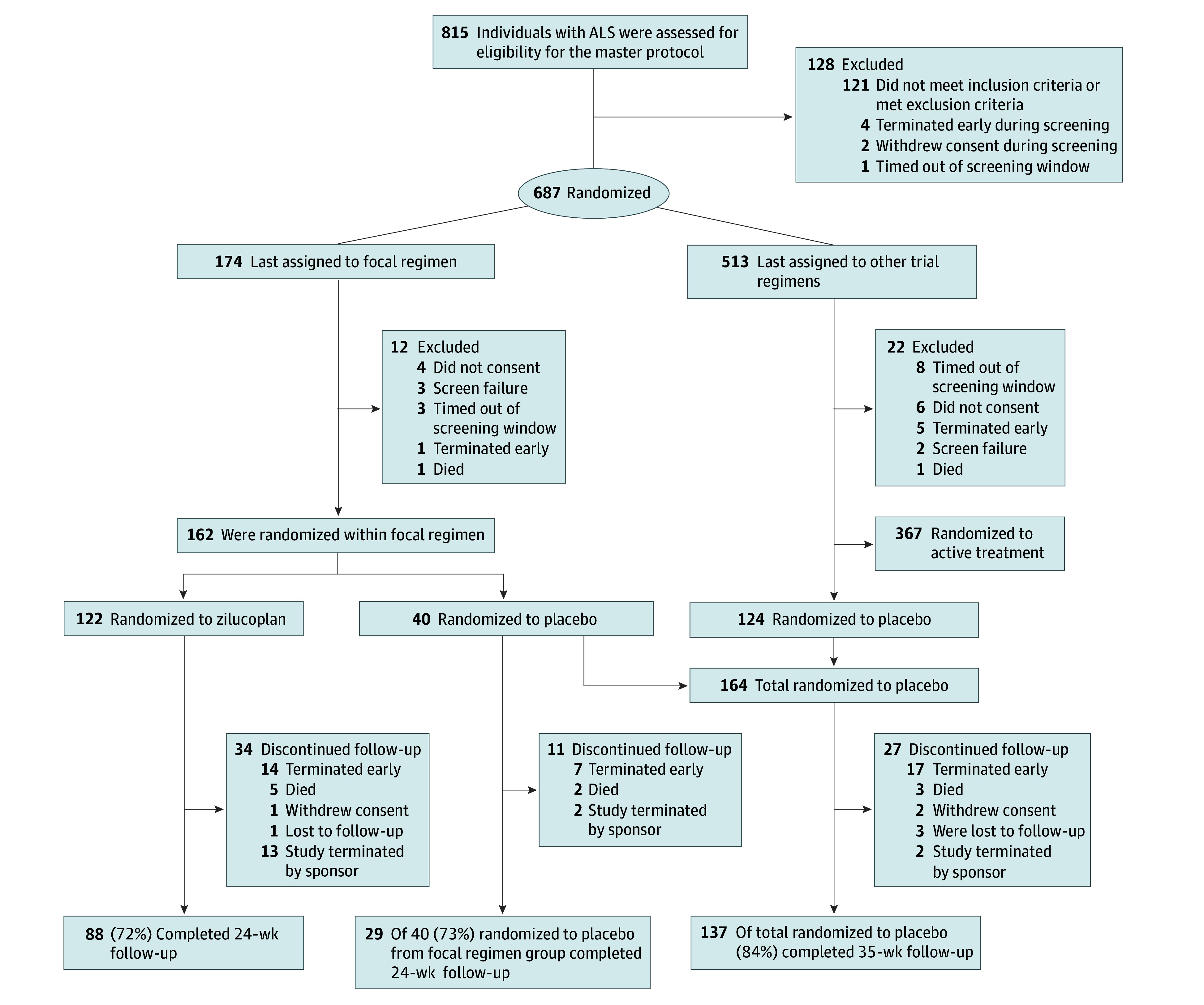
Study Flow Diagram Participants could have multiple reasons for exclusion from the master protocol and could screen for the master protocol more than once. The most common reasons for exclusion were not meeting the criterion for slow vital capacity of at least 50% (66 of 143 [46% of person-visits]), having a clinically significant unstable medical condition (other than amyotrophic lateral sclerosis [ALS]) that would pose a risk to the participant (44 of 143 [31% of person-visits]), and having used investigational treatments for ALS within 5 half-lives (if known) or 30 days (whichever is longer) prior to the master protocol screening visit (24 of 143 [17% of person-visits]). The 29 participants who completed the regimen in the regimen-specific placebo are included in the 137 shared placebo who completed the study.

A total of 174 individuals were screened for regimen A, of whom 162 were randomized (mean [SD] age, 59.6 [11.3] years; 99 [61.1%] male and 63 [38.9%] female) to zilucoplan (122 [75.3%]) or regimen-specific placebo (40 [24.7%]). Of the 155 individuals with race data available, 1 (0.6%) was Asian, 2 (1.3%) were Black or African American, and 152 (98.1%) were White. Of 159 individuals with ethnicity data available, 9 (5.7%) were Hispanic or Latino. An additional 124 participants were randomized to placebo during the course of other concurrently enrolling regimens within the master protocol. Details on randomization and study flow are shown in Figure 1. Baseline demographic and disease characteristics are summarized in Table 1. Most participants were receiving riluzole and/or edaravone at study entry (eTable 1 in Supplement 2).

**Table 1.** Demographic and Baseline Characteristics

Characteristic	Treatment group		
	Zilucoplan (n = 122)	Shared placebo (n = 164)	Regimen-specific placebo (n = 40)
Age, mean (SD), y	60.1 (10.8)	57.8 (11.3)	58.0 (12.7)
Sex, No. (%)			
Male	75 (61.5)	115 (70.1)	24 (60.0)
Female	47 (38.5)	49 (29.9)	16 (40.0)
Race, No. (%)^a^			
American Indian or Alaska Native	0	0	0
Asian	1 (0.8)	2 (1.3)	0
Black or African American	2 (1.7)	6 (3.8)	0
Native Hawaiian or Other Pacific Islander	0	0	0
White	115 (97.5)	151 (94.4)	37 (100)
Other^b^	4 (3.3)	4 (2.4)	3 (7.5)
Ethnicity, No. (%)^c^			
Hispanic or Latino	7 (5.8)	6 (3.7)	2 (5.1)
Not Hispanic or Latino	113 (94.2)	157 (96.3)	37 (94.9)
Bulbar onset, No. (%)	21 (17.2)	29 (17.7)	6 (15.0)
El Escorial criteria, No. (%)			
Clinically definite ALS	44 (36.1)	66 (40.2)	16 (40.0)
Clinically probable ALS	38 (31.1)	40 (24.4)	7 (17.5)
Clinically probable ALS, laboratory supported	35 (28.7)	42 (25.6)	13 (32.5)
Clinically possible ALS	5 (4.1)	16 (9.8)	4 (10.0)
King stage, No. (%)			
1	20 (16.4)	34 (20.7)	9 (22.5)
2	27 (22.1)	39 (23.8)	11 (27.5)
3	33 (27.0)	45 (27.4)	6 (15.0)
4a	3 (2.5)	NA	NA
4a/4b	4 (3.3)	1 (0.6)	NA
4b	35 (28.7)	45 (27.4)	14 (35.0)
Riluzole use, No. (%)	94 (77.0)	126 (76.8)	30 (75.0)
Edaravone use, No. (%)	27 (22.1)	41 (25.0)	10 (25.0)
Time since ALS symptom onset, mean (SD), mo	21.4 (8.3)	21.9 (8.7)	22.4 (9.0)
Time since ALS diagnosis, mean (SD), mo	10.5 (6.4)	10.3 (6.1)	10.5 (6.0)
Slow vital capacity, mean (SD), PPN	76.2 (17.3)	76.0 (16.5)	75.7 (15.6)
Mean (SD) ALSFRS-R^d^			
Total score	34.4 (6.4)	35.1 (6.7)	35.1 (7.1)
Bulbar score	9.7 (2.4)	10.0 (2.3)	10.3 (1.9)
Fine motor score	7.5 (2.9)	7.6 (3.1)	7.8 (3.0)
Gross motor score	7.2 (2.9)	7.3 (3.1)	7.1 (3.3)
Combined motor score	14.7 (4.7)	14.9 (5.4)	14.9 (5.5)
Respiratory score	10.1 (2.4)	10.2 (2.1)	10.0 (2.3)
Prebaseline ALSFRS-R slope, mean (SD), points/mo	0.75 (0.54)	0.66 (0.43)	0.66 (0.48)
Body mass index, mean (SD)^e^	26.8 (5.0)	27.3 (5.0)	28.0 (5.4)
Serum NfL chain protein, median (IQR), pg/mL	77.1 (46.9-117.3)	71.9 (47.6-109.8)	66.9 (50.3-114.2)

Abbreviations: ALS, amyotrophic lateral sclerosis; ALSFRS-R, Amyotrophic Lateral Sclerosis Functional Rating Scale–Revised; NA, not applicable; NfL, neurofilament light; PPN, percent predicted normal.

^a^ Available for 118 participants in the zilucoplan group, 160 in the shared placebo group, and 37 in the regimen-specific placebo group.

^b^ Includes multiracial, not reported, and unknown.

^c^ Available for 120 participants in the zilucoplan group, 163 in the shared placebo group, and 39 in the regimen-specific placebo group.

^d^ Scores range from 11.0 to 47.0, with higher scores indicating better function.

^e^ Calculated as the weight in kilograms divided by the height in meters squared.

### Primary Outcome

**Figure 2.**
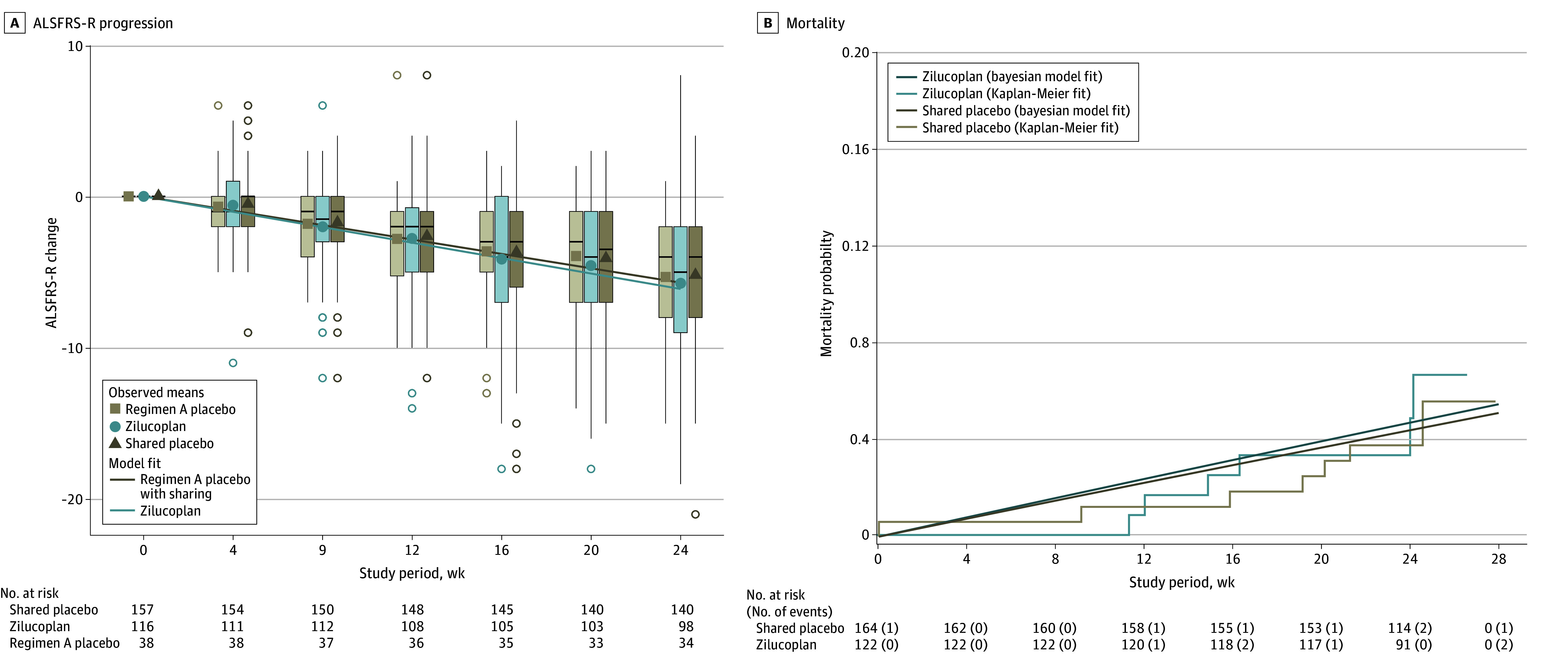
Change in Disease Severity Over 24 Weeks as Measured by Amyotrophic Lateral Sclerosis Functional Rating Scale–Revised (ALSFRS-R) and Survival (Primary Outcome) A, Boxplots of the ALSFRS-R change from baseline over time for the active treatment (light blue), the regimen placebo (light tan), and the shared placebo (dark tan). The bottom of each box represents the 25th percentile of the ALSFRS-R change from baseline values and the top of the box represents the 75th percentile. The horizontal line within each box represents the median value. The whiskers extend to the most extreme data point that is no further than 1.5 times the length of the box away from the box, with outlier values beyond this range shown by open circles. Solid points are the raw mean values at each visit, with different shapes and colors representing active treatment (light blue circles), the regimen placebo group (light tan squares), and the shared placebo group (dark tan triangles). The solid diagonal lines are the model-estimated change in ALSFRS-R over time, adjusting for covariates. The estimate for the regimen placebo group shares information from the placebo groups in other regimens. The numbers below the panel show the number of participants with known ALSFRS-R outcomes at each visit. The summary excludes all ALSFRS-R data from participants who died or had a death-equivalent event (permanent assisted ventilation [PAV]). ALSFRS-R scores range from 11.0 to 47.0, with higher scores indicating better function. B, Kaplan-Meier curves for death or PAV per arm and the model-estimated exponential curves for the active treatment and pooled placebo groups. The numbers below the panel indicate the number of participants exposed and the number of death or PAV events per arm.

The futility criterion was reached at the fourth interim analysis (February 2022). At that point, all 162 participants were randomized in the regimen and 115 (71.0%) had completed week 24 assessment. The posterior probability that the treatment slowed disease progression by at least 10% was 0.05. Participants were instructed to immediately discontinue study dosing, and a final early termination visit was conducted. At the final analysis, the estimated DRR common to ALSFRS-R and survival was 1.08 (95% credible interval [CrI], 0.87-1.31; posterior probability of superiority, 0.24) (Figure 2). The estimated mean slopes of ALSFRS-R total score as modeled by the Bayesian model in the analysis population were –1.11 (95% CrI, −1.27 to −0.94) points/mo for the active treatment group and –1.03 (95% CrI, −1.18 to −0.89) points/mo for the shared placebo group (eTable 2 in Supplement 2). The mortality hazard rates were 0.009 (95% CrI, 0.005-0.010) for the active treatment group and 0.008 (95% CrI, 0.004-0.010) for the shared placebo group (eTable 2 in Supplement 2). Results are consistent for the key supportive joint rank analysis, with mean rank of 134.9 for zilucoplan treatment and 149.9 for shared placebo (*z* = −1.51, 1-sided *P* = .85) and sensitivity analyses regarding the analysis population, including limiting to only the regimen placebo groups, missing data, and modeling assumptions (DRR range, 1.04-1.23). In sensitivity analyses, which allowed for different treatment effects, the DRR for ALSFRS-R was 1.07 (95% CrI, 0.87-1.31) and the DRR for mortality was 1.22 (95% CrI, 0.45-1.95). A summary of the ALSFRS-R scores by group and visit is presented in eTable 3 in Supplement 2.

### Secondary and Exploratory Outcomes

The mean 24-week change in ALSFRS-R total score, hand-held dynamometry upper and lower extremity scores, slow vital capacity, and neurofilament light chain protein levels using repeated-measures analyses did not differ between the zilucoplan and placebo groups (Table 2). The cumulative adjusted hazard ratios for outcome of death or permanent assisted ventilation in the active treatment vs placebo groups are shown in the eTable 4 in Supplement 2. Subgroup analyses for ALSFRS-R slope using a frequentist random slopes model are presented in eTable 5 in Supplement 2. Complement inhibition in plasma was generally 92% or greater at each planned visit as measured by the Wieslab assay and suggests complete complement inhibition (eFigure in Supplement 2).

**Table 2.** Results for Secondary Outcomes Using Repeated-Measures Analysis^a^

End point	Treatment group, 24-wk change estimate (SE)		Difference from shared placebo	
	Zilucoplan	Placebo^b^	Difference (SE) [95% CI]	*P* value
**FAS population**				
ALSFRS-R total score	−6.48 (0.49)	−5.61 (0.41)	−0.87 (0.63) [−2.11 to 0.36]	.17
HHD upper extremity, % change	−33.60 (3.21)	−30.84 (2.68)	−2.77 (4.08) [−10.78 to 5.25]	.50
SVC, % predicted	−9.66 (1.42)	−8.57 (1.16)	−1.09 (1.82) [−4.66 to 2.48]	.55
HHD lower extremity, % change	−17.38 (3.53)	−19.34 (2.93)	1.96 (4.49) [−6.86 to 10.77]	.66
Serum NfL chain protein, % change^c^	−3.19 (NA)	1.90 (NA)	−5.00 (NA) [−15.09 to 6.29]	.37
**ERO population**				
ALSFRS-R total score	−6.52 (0.52)	−5.85 (0.88)	−0.67 (1.01) [−2.66 to 1.33]	.51
HHD upper extremity, % change	−34.82 (3.55)	−28.33 (5.91)	−6.49 (6.81) [−19.94 to 6.96]	.34
SVC, % predicted	−9.76 (1.55)	−6.66 (2.58)	−3.10 (2.97) [−8.96 to 2.76]	.30
HHD lower extremity, % change	−18.12 (3.78)	−23.56 (6.22)	5.44 (7.18) [−8.76 to 19.64]	.45
Serum NfL chain protein, % change^c^	−1.74 (NA)	5.72 (NA)	−7.06 (NA) [−24.49 to 14.40]	.49

Abbreviations: ALSFRS-R, Amyotrophic Lateral Sclerosis Functional Rating Scale–Revised; ERO, efficacy regimen only; FAS, full analysis set; HHD, handheld dynamometer; NA, not applicable; NfL, neurofilament light; SVC, slow vital capacity.

^a^ Models are adjusted for time since symptom onset, prebaseline ALSFRS-R slope, edaravone use at baseline, riluzole use at baseline, and their interactions with visit.

^b^ FAS population includes shared placebo groups (n = 164); ERO population, only regimen-specific placebo group (n = 40).

^c^ Modeled as log-transformed and exponentiated back to the original scale to present results.

### Safety and Tolerability

Nearly all participants in the zilucoplan (116 of 122 [95.1%]) and shared placebo (146 of 163 [89.6%]) groups reported 1 or more treatment-emergent adverse events (TEAEs) during the trial. Most did not lead to modification or interruption of trial drug dosing and were not considered related to treatment (eTable 6 in Supplement 2). The most common TEAEs in the zilucoplan group were falls (39 [32.0%]), muscular weakness (29 [23.8%]), injection site bruising (22 [18.0%]), and neuromyopathy (22 [18.0%]) (eTable 6 in Supplement 2). Among the participants receiving active treatment, totaling 50.21 patient-years of exposure, no *Neisseria* infections were reported. Mean changes in weight, electrocardiography, and safety laboratory values over 24 weeks did not show evidence of differing between groups, except for eosinophil levels, for which a maximum mean change from baseline was observed at week 8 (40 μL in the zilucoplan arm compared with 2 μL in the shared placebo arm [to convert to ×10^9^/L, multiply by 0.001]). Transient elevations of blood eosinophil levels are a known adverse drug reaction to zilucoplan. Fatal TEAEs occurred in 5 participants (4.1%) who received zilucoplan and 4 (2.5%) in the shared placebo group. The most common cause of death overall was respiratory failure, consistent with the natural history of ALS. Serious adverse events occurred in 25 participants (20.5%) in the zilucoplan group and 15 (9.2%) in the shared placebo group. This imbalance was driven by respiratory failure. The incidence rate for respiratory failure in the zilucoplan arm was 19.76 per 100 patient-years. Twelve participants (9.8%) in the zilucoplan group prematurely discontinued trial drug owing to TEAEs, compared with 11 (6.7%) in the shared placebo group.

### Impact of Early Stopping on Trial Operations

**Figure 3.**
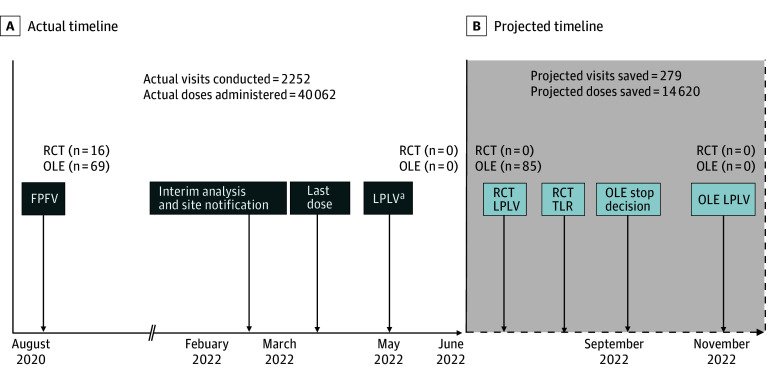
Study Timeline A, The actual timeline for trial completion after the futility criterion was met at the February 2022 interim analysis. B, The projected timeline for trial completion if interim analyses did not occur and all participants completed the full 24 weeks of the randomized placebo-controlled portion of the regimen. Using the projected dates of trial completion, the sum of all expected visits that would have occurred between the actual and projected last participant last visit (LPLV) was calculated using individual participant visit schedules generated from the electronic data capture system. FPFV indicates first participant first visit; OLE, open-label extension; RCT, randomized clinical trial; and TLR, top line results. ^a^Per the regimen specific appendix, the final study visit for a participant occurred 40 plus or minus 3 days after discontinuing investigational product treatment.

As the futility criterion was reached at the February 2022 interim analysis, an analysis was performed to determine the additional number of study visits that would have been conducted and additional study drug doses that would have been administered if the interim analysis had not been conducted and all participants completed the full 24-week study period. Of note, participants who completed the randomized clinical trial were eligible for enrollment in an open-label extension trial evaluating the long-term effects of zilucoplan. The open-label extension was stopped at the same time as the randomized clinical trial. Actual trial operational dates that occurred following the February 2022 interim analysis, including date of last participant dose and LPLV, are included in eTable 7 in Supplement 2. Projected dates of trial completion for all participants completing week 24 are presented in eTable 7 in Supplement 2. Between the actual LPLV following the interim analysis (May 5, 2022) and the projected LPLV for the full 24-week follow-up (October 24, 2022), an additional 29 study visits in the randomized placebo-controlled portion of the study and 250 study visits in the open-label extension would have occurred. Comparison of the actual vs projected timelines is included in Figure 3. At the time the futility criterion was met, 75 participants were receiving study drug in the randomized placebo-controlled portion or the open-label extension of the trial. As zilucoplan is administered once daily, an additional 14 620 doses of active or placebo would have been administered to participants if no discontinuations occurred.

## Discussion

In this randomized clinical trial, treatment with zilucoplan did not affect disease progression in participants with ALS as assessed by the ALSFRS-R total score and survival (shared-parameter model) over a period of 24 weeks. Secondary outcomes did not differ significantly between the 2 groups.

Similar to prior trials of zilucoplan in people with generalized myasthenia gravis,^7^ immune-mediated necrotizing myopathy,^14^ and paroxysmal nocturnal hemoglobinuria,^15^ complete complement inhibition in plasma was observed in our study. Thus, the lack of efficacy of zilucoplan in ALS cannot be attributed to a failure of achieving its intended effect. While preclinical data point to complement activation as one of the pathways implicated in ALS, the study results, in conjunction with the recent negative studies of ravulizumab^16^ and pegcetacoplan^17^ in ALS, questions exist about the role of complement inhibition as a treatment strategy for ALS. Of note, preclinical evidence for putative therapeutics in ALS is often not corroborated in clinical trials, which could be due to a variety of reasons, including animal model limitations, clinical disease heterogeneity, or other factors. Importantly, there is no consensus on what constitutes sufficient preclinical evidence to advance investigational products to human studies.

Complement inhibition is an effective therapeutic strategy in other neurological diseases, such as myasthenia gravis. In this disorder, approved treatment options include 3 complement C5 inhibitors: zilucoplan,^7^ eculizumab,^18^ and ravulizumab.^19^ The data reported herein add to the extant literature on the safety of zilucoplan. Similar to other zilucoplan studies, the safety profile was dominated by events consistent with the underlying disease, and no unexpected safety signals for zilucoplan were identified. The most common cause of death in our study was respiratory failure, with an incidence rate for respiratory failure in the zilucoplan arm of 19.76 per 100 patient-years; this was consistent with the natural disease course of ALS with a background rate of 23.19 per 100 patient-years observed in patients with ALS in a cohort analysis of US claims data in the period between June 30, 2010, and June 30, 2021, in an unpublished internal study conducted by UCB.

### Strengths and Limitations

Despite the disappointing efficacy results of this study, there are several important strengths to highlight. The platform trial approach allowed for efficiency across treatment regimens through shared trial infrastructure and use of a shared placebo group. In addition, this regimen demonstrated the value of the interim analysis approach. Interim analyses are conducted within the master protocol with the potential for a regimen to stop early for futility if prespecified criteria are met. The interim analysis for zilucoplan provided study results approximately 5 months sooner than the initially projected time of study completion. This approach contributed to several operational efficiencies, including less participant exposure to zilucoplan, fewer study visits conducted, and reallocation of resources to other studies. By stopping the study for futility, study participants were spared 279 trial visits and a total of 14 620 doses of investigational product, reducing unnecessary burden, and potentially allowing participants to enroll in other research studies.

This study also has several limitations. Complement activation and inhibition pathways are complex, and it is possible that interventions further upstream in the complement pathway could have different treatment effects. Furthermore, ALS is a heterogeneous disease. It is possible that complement activation may play different roles at different stages of the disease or in specific subgroups. This trial enrolled a relatively broad population with ALS and was not powered to identify subsets of participants who could benefit from treatment.

## Conclusions

In this randomized clinical trial of zilucoplan, treatment had a low likelihood of efficacy for ALS. No unexpected treatment-related risks were identified. The prespecified futility analyses reduced risk and burden to study participants and the biomarker data provided confirmation of target engagement. This study provides valuable safety and tolerability data for zilucoplan and this mechanism of action.

## Notes

Supplement 1.
Trial Protocol and Statistical Analysis Plan

Supplement 2. **eTable 1.** Use of Riluzole and Edaravone at Baseline and During Trial by Group **eTable 2.** Bayesian Shared Parameter Model Results: Primary Analysis (FAS Sample) **eTable 3.** Distribution of the ALSFRS-R by Groups and Visits **eTable 4.** Cox Proportional Hazards Regression on Survival to Death or PAV **eTable 5.** Subgroup Results From the Repeated Measures Model **eTable 6.** Adverse Events **eTable 7.** Data Tables **eFigure.** Mean Complement Activity as Assessed by the Alternative Complement Pathway Specific Wieslab Assay to Week 24 **eMethods.** Design and Participants
eReference

Supplement 3. **Nonauthor Collaborators.** The HEALEY ALS Platform Trial Study Group Members

Supplement 4.
Data Sharing Statement
