# Supplementary material for: Efficacy and Safety of Zilucoplan in Amyotrophic Lateral Sclerosis: A Randomized Clinical Trial
Source: JAMA Netw Open. 2025 Feb 17;8(2):e2459058. doi: 10.1001/jamanetworkopen.2024.59058 (PMC11833520; doi:10.1001/jamanetworkopen.2024.59058)
Supplement: Supplement 2. — eTable 1. Use of Riluzole and Edaravone at Baseline and During Trial by Group eTable 2. Bayesian Shared Parameter Model Results: Primary Analysis (FAS Sample) eTable 3. Distribution of the ALSFRS-R by Groups and Visits eTable 4. Cox Proportional Hazards Regression on Survival to Death or PAV eTable 5. Subgroup Results From the Repeated Measures Model eTable 6. Adverse Events eTable 7. Data Tables eFigure. Mean Complement Activity as Assessed by the Alternative Complement Pathway Specific Wieslab Assay to Week 24 eMethods. Design and Participants eReference [file jamanetwopen-e2459058-s002.pdf]

Supplementary Online Content

Paganoni S, Fournier CN, Macklin EA, et al; HEALEY ALS Platform Trial Study Group. Efficacy and safety of zilucoplan in amyotrophic lateral sclerosis: a randomized clinical trial. *JAMA Netw Open*. 2025;8(2):e2459058. doi:10.1001/jamanetworkopen.2024.59058

eTable 1. Use of Riluzole and Edaravone at Baseline and During Trial by Group

eTable 2. Bayesian Shared Parameter Model Results: Primary Analysis (FAS Sample)

eTable 3. Distribution of the ALSFRS-R by Groups and Visits

eTable 4. Cox Proportional Hazards Regression on Survival to Death or PAV

eTable 5. Subgroup Results From the Repeated Measures Model

eTable 6. Adverse Events

eTable 7. Data Tables

eFigure. Mean Complement Activity as Assessed by the Alternative Complement Pathway Specific Wieslab Assay to Week 24

eMethods. Design and Participants

eReference

This supplementary material has been provided by the authors to give readers additional information about their work.

© 2025 Paganoni S et al. *JAMA Network Open*.

eTable 1. Use of Riluzole and Edaravone at Baseline and During Trial by Group

Characteristic		Zilucoplan (N = 122)	Shared Placebo[a] (N = 163)	Regimen-specific Placebo (N = 40)
**ALS associated medications**				
Riluzole - N (%)				
	At baseline	94 (77.0%)	125 (76.2%)	30 (75.0%)
	Initiated during trial	1 (0.8%)	1 (0.6%)	1 (2.5%)
Edaravone - N (%)				
	At baseline	27 (22.1%)	40 (24.4%)	10 (25.0%)
	Initiated during trial	0 (0.0%)	0 (0.0%)	0 (0.0%)

[a] One participant in the shared placebo was randomized but never initiated drug, therefore is excluded from the safety population

© 2025 Paganoni S et al. *JAMA Network Open*.

eTable 2. Bayesian Shared Parameter Model Results: Primary Analysis (FAS Sample)

Parameter	Median	Mean (SD)	95% CI	Pr(DRR<1)	Pr(DRR<0.9)
**DRR**					
Function and Mortality	1.07	1.08 (0.11)	(0.87, 1.31)	0.24	0.05
**ALSFRS-R Slopes (points per month)**					
Regimen A placebo w/ sharing	-1.03	-1.03 (0.07)	(-1.18, -0.89)		
Zilucoplan	-1.11	-1.11 (0.08)	(-1.27, -0.94)		
**Mortality Event Rate (events per month)**					
Shared placebo	0.008	0.008 (0.0023)	(0.004, 0.01)		
Zilucoplan	0.009	0.009 (0.0025)	(0.005, 0.01)		
**Covariates**					
Months since onset	1.07	1.07 (0.05)	(0.98, 1.17)		
Pre-baseline slope	1.42	1.43 (0.09)	(1.27, 1.61)		
Edaravone use	1.19	1.20 (0.10)	(1.01, 1.42)		
Riluzole use	0.97	0.97 (0.09)	(0.81, 1.16)		

CI – credible interval

© 2025 Paganoni S et al. *JAMA Network Open*.

eTable 3. Distribution of the ALSFRS-R by Groups and Visits

	Zilucoplan		Shared Placebo		Regimen Placebo	
**Visit**	**n**	**mean (SD)**	**n**	**mean (SD)**	**n**	**mean (SD)**
Baseline	122	34.4 (6.4)	164	35.1 (6.7)	40	35.1 (7.1)
Week 4	117	33.8 (6.8)	160	34.5 (7.0)	40	34.4 (7.9)
Week 8	117	32.3 (7.4)	155	33.4 (7.4)	39	33.4 (8.0)
Week 12	113	31.2 (8.6)	152	32.7 (7.9)	38	32.5 (8.9)
Week 16	109	30.2 (9.0)	148	31.8 (8.3)	36	32.3 (8.8)
Week 20	105	29.6 (9.2)	143	31.2 (8.6)	34	31.9 (9.2)
Week 24	100	28.9 (9.3)	143	30.2 (8.7)	35	30.3 (9.5)

© 2025 Paganoni S et al. *JAMA Network Open*.

eTable 4. Cox Proportional Hazards Regression on Survival to Death or PAV

	Observed # events/ # population		Hazard Ratio	95% CI	p-value
	Zilucoplan	Placebo			
Zilucoplan vs Shared Placebo	4/122	9/162	0.86	0.31, 2.31	0.76
Zilucoplan vs Regimen A Placebo	4/122	2/39	1.07	0.26, 7.22	0.93

PAV – Permanent assisted ventilation

95% CI: 95% Confidence Interval by Profile Likelihood
*Models adjusted for age, sex, time since symptom onset, pre-baseline ALSFRS-R slope, riliuzole use at baseline, edaravone use at baseline

© 2025 Paganoni S et al. *JAMA Network Open*.

eTable 5. Subgroup Results From the Repeated Measures Model

Models adjusted for time since symptom onset, pre-baseline ALSFRS-R slope, edaravone use at baseline, riluzole use at baseline and their interactions with visit.

Category	Zilucoplan Slope (change/month) LS mean (95% CI)	Shared Placebo Slope (change/month) LS mean (95% CI)	Plot (Favors Placebo ← → Favors Treatment)	Difference	p-value
**All Participants**	-1.18 (-1.35,-1.01)	-1.02 (-1.16,-0.87)		-0.16 (-0.38,0.06)	NA
**Riluzole Use**					
N	-1.46 (-1.81,-1.11)	-0.90 (-1.20,-0.60)		-0.56 (-1.01,-0.10)	0.051
Y	-1.09 (-1.29,-0.90)	-1.05 (-1.21,-0.89)		-0.04 (-0.29,0.21)	NA
**Edaravone Use**					
N	-1.18 (-1.37,-0.99)	-0.96 (-1.13,-0.79)		-0.22 (-0.47,0.03)	0.35
Y	-1.18 (-1.53,-0.83)	-1.21 (-1.51,-0.91)		0.03 (-0.43,0.49)	NA
**Riluzole & Edaravone Use**					
Neither	-1.38 (-1.74,-1.02)	-0.82 (-1.14,-0.51)		-0.56 (-1.03,-0.08)	NA
Riluzole Only	-1.10 (-1.33,-0.88)	-1.01 (-1.20,-0.82)		-0.09 (-0.38,0.20)	NA
Edaravone Only	-2.22 (-3.59,-0.85)	-1.44 (-2.45,-0.44)		-0.78 (-2.48,0.92)	NA
Both	-1.09 (-1.45,-0.73)	-1.17 (-1.48,-0.87)		0.08 (-0.39,0.56)	NA
**Age Group**					
< 65	-1.15 (-1.37,-0.93)	-1.04 (-1.21,-0.87)		-0.11 (-0.39,0.16)	0.54
>= 65	-1.22 (-1.48,-0.96)	-0.96 (-1.23,-0.69)		-0.26 (-0.63,0.12)	NA

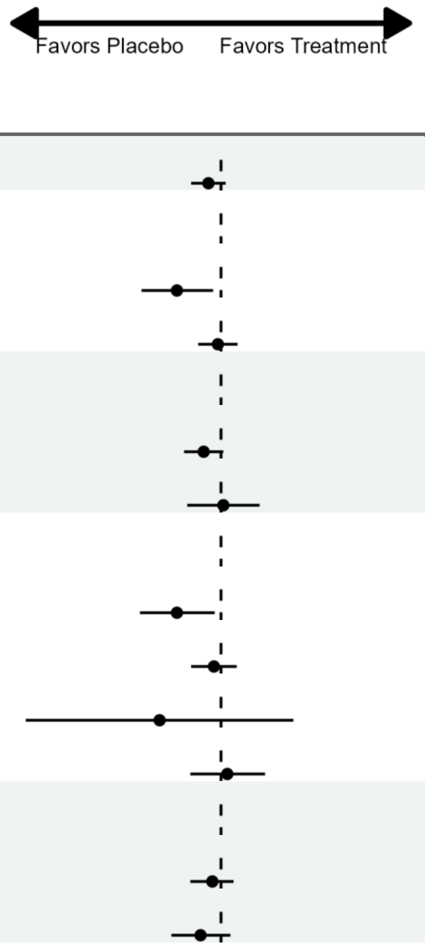

© 2025 Paganoni S et al. *JAMA Network Open*.

Category	Zilucoplan Slope (change/month) LS mean (95% CI)	Shared Placebo Slope (change/month) LS mean (95% CI)	Plot	Difference	p-value
**Sex**					
F	-1.36 (-1.63,-1.09)	-1.11 (-1.37,-0.84)		-0.25 (-0.63,0.12)	0.49
M	-1.07 (-1.28,-0.86)	-0.98 (-1.15,-0.81)		-0.09 (-0.36,0.18)	NA
**Race**					
Other	-1.11 (-1.79,-0.42)	-0.84 (-1.37,-0.31)		-0.27 (-1.13,0.59)	0.79
White	-1.18 (-1.36,-1.01)	-1.03 (-1.18,-0.88)		-0.15 (-0.38,0.08)	NA
**Ethnicity**					
Hispanic or Latino	-0.73 (-1.41,-0.04)	-0.91 (-1.69,-0.12)		0.18 (-0.86,1.22)	0.48
Not Hispanic or Latino	-1.22 (-1.40,-1.05)	-1.02 (-1.17,-0.88)		-0.20 (-0.42,0.03)	NA
**Weight Group**					
< 56 kg	-1.65 (-2.23,-1.08)	-1.13 (-1.79,-0.46)		-0.53 (-1.40,0.35)	NA
56 to <77 kg	-1.33 (-1.60,-1.05)	-1.06 (-1.29,-0.82)		-0.27 (-0.63,0.09)	NA
77 to <150 kg	-1.02 (-1.24,-0.79)	-0.99 (-1.18,-0.80)		-0.03 (-0.32,0.26)	NA
**BMI Group**					
< 18.5 kg/m^2	0.26 (-1.50,2.02)	-1.34 (-2.36,-0.33)		1.60 (-0.44,3.64)	NA
18.5 to < 25 kg/m^2	-1.49 (-1.78,-1.20)	-1.08 (-1.32,-0.83)		-0.41 (-0.79,-0.04)	NA
25 to < 30 kg/m^2	-1.07 (-1.32,-0.83)	-0.95 (-1.18,-0.73)		-0.12 (-0.46,0.21)	NA
30 to < 40 kg/m^2	-1.06 (-1.45,-0.67)	-1.11 (-1.41,-0.81)		0.05 (-0.44,0.54)	NA
>= 40 kg/m^2	-0.62 (-1.50,0.26)	-0.06 (-1.08,0.95)		-0.56 (-1.90,0.78)	NA

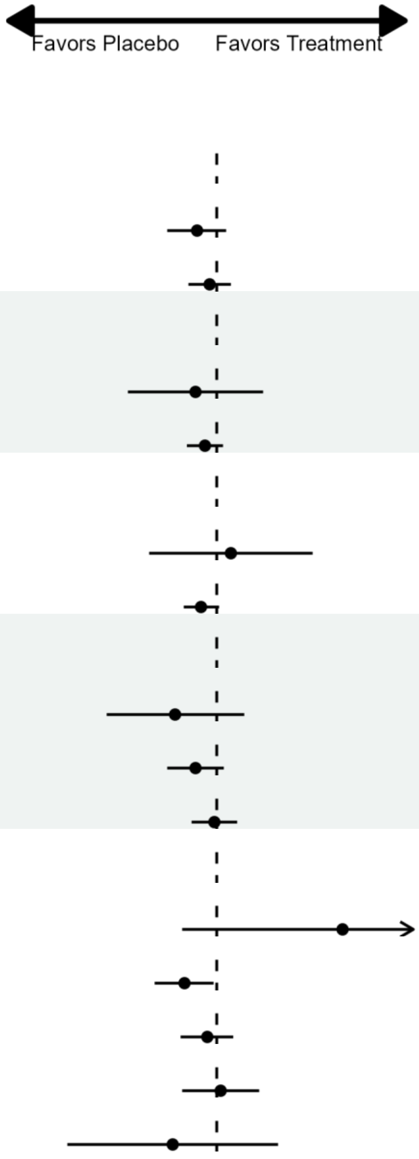

© 2025 Paganoni S et al. *JAMA Network Open*.

Category	Zilucoplan Slope (change/month) LS mean (95% CI)	Shared Placebo Slope (change/month) LS mean (95% CI)	Plot (Favors Placebo / Favors Treatment)	Difference	p-value
**Chronic Kidney Disease**					
Stage 1 or better	-1.16 (-1.37,-0.94)	-1.05 (-1.23,-0.88)		-0.10 (-0.38,0.17)	NA
Stage 2	-1.25 (-1.53,-0.96)	-0.94 (-1.20,-0.68)		-0.31 (-0.70,0.08)	NA
Stage 3	-0.68 (-1.95,0.58)	-0.99 (-2.77,0.80)		0.30 (-1.89,2.49)	NA
**Time Since Symptom Onset**					
< 18 months	-1.30 (-1.64,-0.96)	-1.02 (-1.32,-0.72)		-0.28 (-0.66,0.10)	0.46
>= 18 months	-1.12 (-1.35,-0.89)	-1.01 (-1.23,-0.80)		-0.11 (-0.38,0.16)	NA
**El Escorial Definite & Time Since Symptom Onset**					
Definite and < 18 months	-1.80 (-2.29,-1.31)	-1.30 (-1.74,-0.86)		-0.50 (-1.13,0.12)	0.25
Not Definite and < 18 months	-1.10 (-1.28,-0.92)	-0.98 (-1.13,-0.83)		-0.12 (-0.35,0.11)	NA
**Baseline Symptom Severity Group**					
All ALSFRS-R Items >= 2	-1.09 (-1.45,-0.73)	-0.93 (-1.18,-0.67)		-0.17 (-0.60,0.26)	0.94
>=1 ALSFRS-R Items < 2	-1.22 (-1.43,-1.02)	-1.08 (-1.27,-0.88)		-0.15 (-0.40,0.11)	NA
**Baseline NFL Group**					
NfL < Median	-0.83 (-1.06,-0.59)	-0.96 (-1.16,-0.77)		0.14 (-0.16,0.43)	0.01
NfL >= Median	-1.54 (-1.78,-1.30)	-1.08 (-1.29,-0.88)		-0.46 (-0.76,-0.15)	NA
**Fast vs. Slow Progressors**					
dFRS < Median	-1.14 (-1.39,-0.89)	-0.97 (-1.18,-0.76)		-0.17 (-0.47,0.14)	0.99

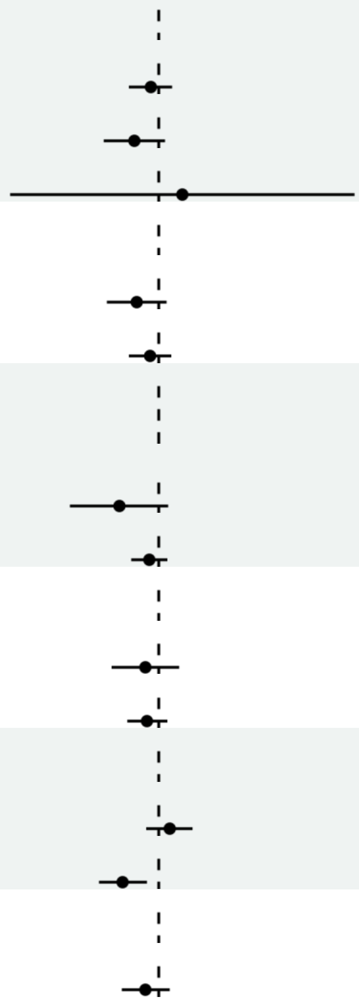

© 2025 Paganoni S et al. *JAMA Network Open*.

Category	Zilucoplan Slope (change/month) LS mean (95% CI)	Shared Placebo Slope (change/month) LS mean (95% CI)	Plot<br>Favors Placebo ⟷ Favors Treatment	Difference	p-value
dFRS >= Median	-1.24 (-1.53,-0.96)	-1.08 (-1.32,-0.84)	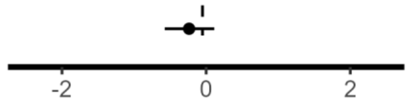	-0.17 (-0.48,0.15)	NA

© 2025 Paganoni S et al. *JAMA Network Open*.

eTable 6. Adverse Events

Treatment Emergent Adverse Events (TEAE)	Zilucoplan (N=122)	Shared placebo[a] (n = 163)	Regimen-specific Placebo (n = 40)
Any TEAE	116 (95.1%), 779	146 (89.6%), 882	37 (92.5%), 220
Mild	40 (32.8%), 591	68 (41.7%), 667	18 (45.0%), 160
Moderate	44 (36.1%), 146	53 (32.5%), 163	12 (30.0%), 40
Severe	32 (26.2%), 42	25 (15.3%), 47	7 (17.5%), 20
Serious TEAEs	25 (20.5%), 31	15 (9.2%), 21	3 (7.5%), 4
Treatment-Related Serious TEAE	4 (3.3%), 5	2 (1.2%), 3	0 (0.0%), 0
Deaths	5 (4.1%), 5	4 (2.5%), 4	2 (5.0%), 2
TEAEs Leading to Study Drug Withdrawal	12 (9.8%), 15	11 (6.7%), 17	2 (5.0%), 3
TEAEs Leading to Study Drug Interruption	6 (4.9%), 12	12 (7.4%), 24	5 (12.5%), 14
TEAEs Leading to Study Drug Reduction	0 (0.0%), 0	5 (3.1%), 12	0 (0.0%), 0
**TEAEs with ≥5% incidence in either group**			
**MedDRA Preferred Term**			
Fall	39 (32.0%), 76	43 (26.4%), 79	9 (22.5%), 18
Muscular weakness	29 (23.8%), 45	45 (27.6%), 67	8 (20.0%), 11
Injection site bruising	22 (18.0%), 24	6 (3.7%), 7	6 (15.0%), 7
Neuromyopathy	22 (18.0%), 27	22 (13.5%), 33	5 (12.5%), 14
Fatigue	17 (13.9%), 18	30 (18.4%), 33	5 (12.5%), 5
Injection site pain	18 (14.8%), 23	2 (1.2%), 2	2 (5.0%), 2
Headache	14 (11.5%), 21	18 (11.0%), 22	4 (10.0%), 4
Constipation	14 (11.5%), 16	19 (11.7%), 23	3 (7.5%), 5
Dysphagia	12 (9.8%), 14	18 (11.0%), 22	4 (10.0%), 4
Dizziness	12 (9.8%), 14	10 (6.1%), 13	2 (5.0%), 3
Nausea	10 (8.2%), 12	14 (8.6%), 16	3 (7.5%), 3
Decreased appetite	6 (4.9%), 6	12 (7.4%), 13	5 (12.5%), 6
Diarrhoea	8 (6.6%), 9	12 (7.4%), 14	3 (7.5%), 3
Urinary tract infection	6 (4.9%), 7	10 (6.1%), 10	5 (12.5%), 5

© 2025 Paganoni S et al. *JAMA Network Open*.

Dysarthria	9 (7.4%), 9	11 (6.7%), 13	2 (5.0%), 2
Respiratory failure	10 (8.2%), 10	3 (1.8%), 3	1 (2.5%), 1
Oedema peripheral	9 (7.4%), 10	12 (7.4%), 15	1 (2.5%), 3
Cough	8 (6.6%), 9	4 (2.5%), 4	1 (2.5%), 1
Dyspnoea	7 (5.7%), 8	10 (6.1%), 11	2 (5.0%), 2
Pruritus	7 (5.7%), 7	4 (2.5%), 6	2 (5.0%), 3
COVID-19	5 (4.1%), 5	11 (6.7%), 11	4 (10.0%), 4
Contusion	7 (5.7%), 7	7 (4.3%), 10	2 (5.0%), 2
Anxiety	7 (5.7%), 8	8 (4.9%), 8	1 (2.5%), 1
Tension headache	6 (4.9%), 6	10 (6.1%), 15	2 (5.0%), 3
Rash	7 (5.7%), 7	4 (2.5%), 4	1 (2.5%), 1
Pain in extremity	5 (4.1%), 9	9 (5.5%), 10	2 (5.0%), 2
Arthralgia	5 (4.1%), 5	6 (3.7%), 6	2 (5.0%), 2
Hypertension	5 (4.1%), 5	4 (2.5%), 4	2 (5.0%), 2
Salivary hypersecretion	4 (3.3%), 4	9 (5.5%), 9	3 (7.5%), 3
Injection site reaction	4 (3.3%), 4	2 (1.2%), 2	2 (5.0%), 2
Post-traumatic pain	3 (2.5%), 3	6 (3.7%), 6	2 (5.0%), 2
Tinnitus	1 (0.8%), 2	3 (1.8%), 3	2 (5.0%), 2
Hepatic enzyme increased	1 (0.8%), 1	2 (1.2%), 2	2 (5.0%), 2
Traumatic haematoma	1 (0.8%), 1	2 (1.2%), 2	2 (5.0%), 2
Vomiting	1 (0.8%), 1	6 (3.7%), 6	2 (5.0%), 2
Hypokalaemia	0 (0.0%), 0	2 (1.2%), 2	2 (5.0%), 2

Presented as number of participants, (%), number of events

[a]One participant in the shared placebo, was randomized but never initiated drug, therefore is excluded from the safety population

© 2025 Paganoni S et al. *JAMA Network Open*.

eTable 7. Data Tables

Actual Data Tables

Regimen A Actual Dates

Milestone	*Date*
RGA RCT FPFV	8/17/2020
Notification of Interim Futility Analysis to Sites	2/28/2022
RGA RCT Date of Last Dose	3/9/2022
RGA RCT LPLV	5/2/2022
RGA OLE FPFV	2/2/2021
RGA OLE Date of Last Dose	3/22/2022
RGA OLE LPLV	5/4/2022

Regimen A Operational Data from EDC

Active participants in RCT as of 2/28/22	16
Active participants in OLE as of 2/28/22	69
Total Visits Completed in RCT	1451
Total Visits Completed in OLE	801
Total Doses Administered in RCT	24,460
Total Doses Administered in OLE	15,602

Projected Data Tables

Regimen A Projected Dates

Milestone	*Date*
RGA LPLV	*6/6/2022*
RCT DBL (6/6/2022 + 8 weeks)	*8/1/2022*
TLR (RCT DBL+ 5 weeks)	*9/5/2022*
Stop Decision (TLD + 1 week)	*9/12/2022*
OLE LPLV Target (stop decision + 6 weeks)	*10/24/2022*

Estimated Operational Data if RGA Futility Analysis not Conducted

Additional RCT Visits to have Occurred	29
Additional OLE Visits to have Occurred	250
Total Visits Saved	279
Additional Daily Doses to have been Administered	14,620

© 2025 Paganoni S et al. *JAMA Network Open*.

eFigure. Mean Complement Activity as Assessed by the Alternative Complement Pathway Specific Wieslab Assay to Week 24

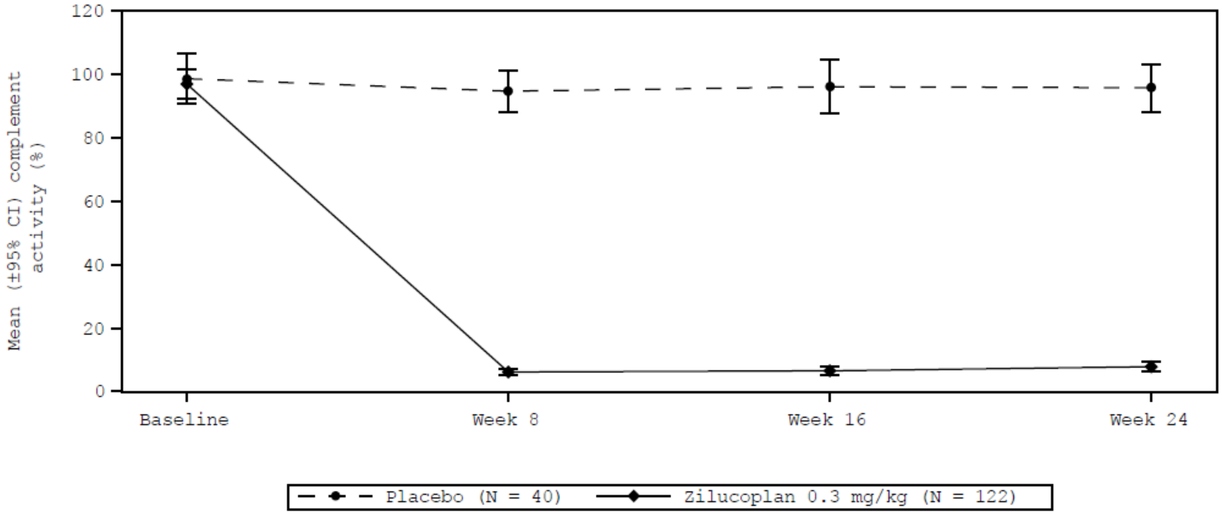

CI - Confidence Interval; Note: inlcudes participants on treatment only

© 2025 Paganoni S et al. *JAMA Network Open*.

eMethods. Design and Participants

Trial Design and Oversight

The HEALEY ALS Platform Trial is a novel, efficient, perpetual design, platform trial designed to test multiple investigational products against placebo in parallel. In this platform trial, each new investigational product is tested as a regimen specific appendix, or "Regimen" to the master protocol. Zilucoplan was evaluated in Regimen A.

Trial Participants

Those who were eligible for the HEALEY ALS Platform Trial were adults with a diagnosis of clinically possible, probable, lab-supported probable, or definite ALS defined by revised El Escorial criteria, disease duration ≤ 36 months, a vital capacity ≥ 50% predicted for age, height, and sex, the ability to swallow pills and liquids, and either no use of riluzole and/or edaravone or stable dosing of riluzole and/or edaravone for more than 30 days or one cycle, respectively. Full inclusion and exclusion criteria can be viewed in the trial protocol available as a Supplement.

Randomization techniques

Both the randomization to regimen, which was not blinded, and the randomization to active drug or placebo within a regimen, which was blinded to both participant and investigator, were done using as pre-determined randomization schedule. In order to ensure balance in regimens and treatment groups by current medication use, both randomization schedules were stratified by baseline use of riluzole and edaravone (4 strata total).

Random assignment to a regimen was done. Random assignment to a regimen within a stratum uses a block urn design[1] with one instance of each regimen in a given block and modified to remove from consideration any regimens to which the participant cannot be assigned (termed "unavailable regimens").

Within each strata a blocking schema was used to create the randomization schedule. Blocks were of size 4 (3 for zilucoplan and 1 for placebo). Randomization schedules were created using SAS software and validated to ensure no discernable patterns were seen before implementation.

Trial Interventions and Procedures

Eligible participants were randomized 3:1 ratio to receive zilucoplan, or matching placebo, with stratification for edaravone and/or riluzole use. Active drug (zilucoplan 0.3 mg/kg) and placebo were provided for subcutaneous (SC) admin for daily dosing. The primary analysis compared those receiving active zilucoplan, to placebo. Trial duration was 24 weeks. Clinic visits were conducted at baseline, Weeks 4, 8, 16 and 24, and telephone visits at Weeks 2, 12, and 20, with a final telephone follow-up at week 28. Participants who completed the randomized, double-blind trial were eligible for enrollment in an open-label extension (OLE) evaluating the long-term effects of zilucoplan (NCT04436497).

Outcomes

The primary efficacy outcome was change in the rate of disease progression, as measured by ALS Functional Rating Scale – Revised (ALSFRS-R) and survival, from baseline through 24 weeks. The ALSFRS-R consists of 12 items across four subdomains of bodily function (i.e., bulbar, fine motor, gross motor, and breathing), with each item being scored on an ordinal scale (0 = total loss of function, 4 = no loss of function, maximum 48, lower scores indicating greater functional difficulty). The scale, validated for administration in person or by telephone, has shown high inter- and intra-rater reliability.

Secondary clinical efficacy outcomes included the rate of decline in isometric muscle strength as measured by Hand-Held Dynamometry (HHD) and the rate of decline in slow vital capacity (SVC). HHD was also assessed as time to loss of strength, defined as the time from baseline visit to the first post-baseline occurrence of a muscle with a strength recording of 0 among those muscles that were non-zero at baseline.

© 2025 Paganoni S et al. *JAMA Network Open*.

Additional prespecified outcomes included rates of decline in ALSFRS-R subdomains; quantitative voice characteristics as measured by Aural Analytics (maximum phonation time, pause rate, breathy vocal quality, pitch instability, regulation of voicing, articulatory precision, speaking rate, articulation rate, and monotonicity); changes in biofluid markers of neurodegeneration and neuromuscular degeneration, including neurofilament light chain protein; change in respiratory function as assessed by home spirometry; and change in complement pathway biomarker levels in blood (as assessed by the Wieslab alternative complement pathway assay). Death or death-equivalent events (tracheostomy or permanent assisted ventilation more than 22 hours daily for more than 7 consecutive days]),[7] tracheostomy placement, and hospitalization events (excluding elective surgeries) were captured. A hierarchy was prepared for secondary outcomes for inference testing. The full statistical analysis plan is provided as a Supplement.

Isometric muscle strength of seven upper and four lower extremity muscle groups was assessed using HHD, with three trials of each muscle group. Respiratory muscle function was assessed by SVC, measured in an upright position for at least three trials per assessment or for up to five trials when the highest and second highest of the first three measurements differed by 10% or more. SVC volumes were standardized to PPN based on age, sex, and height. The highest recorded SVC score from all attempts was utilized for analysis.

Safety was mainly assessed via documentation of treatment-emergent adverse events (TEAEs). Symptoms of ALS progression, including those consistent with disease progression, were recorded as TEAEs. Any worsening of a disease progression measure that was being recorded and analyzed separately (i.e., ALSFRS-R, HHD, and SVC) was not recorded as a TEAE. Safety was additionally assessed using laboratory assays, electrocardiograms, suicidality, and changes in vital signs.

Pharmacodynamic assessment

Blood samples for pharmacodynamic analysis (WIESLAB® *Complement System Alternative Pathway - Research Use Only;* part no: COMPL AP330RUO) were collected prior to the dose at baseline, Week 8, Week 16 and Week 24 visits.

Statistical analysis methods

Primary Analysis

The primary analysis is a Bayesian shared parameter model of function and survival that provides an integrated estimate of the increase or decrease in the rate of disease progression on treatment relative to control. The analysis model has components for each endpoint that are linked through an integrated estimate of disease slowing in treatment relative to control across the two endpoints (denoted as the disease rate ratio, or DRR). This composite variable strategy fully incorporates effects of treatment on the intercurrent event of death. The DRR measures the ratio of ALSFRS-R slopes within the functional component or the hazard ratio within the survival component with values less than 1 indicating slowing in disease progression This parameter can be interpreted as the slowing in the rate of disease progression measured across both endpoints. A DRR of 0.50 corresponds to a 50% slowing in the rate of disease progression. If a participant in the control group would be expected to decrease 6 points over 6 months, then under a DRR of 0.50, they would be expected to reach the same milestone (ALSFRS-R decrease of 6 points) over 12 months on treatment. Similarly for survival, if they were expected to survive for a median time of 9 months on control, under the treatment they would be expected to survive for 18 months. In the functional component, ALSFRS-R is measured for those who have survived using a linear repeated measure model that adjusts for baseline covariates and accommodates potential differences in the shared control across regimens. The survival component is measured through an exponential proportional hazards model. The shared treatment effect between the ALSFRS-R and mortality components allows the participants who were lost to follow-up due to mortality to inform treatment effect estimates beyond their censored ALSFRS-R longitudinal data. The degree to which treatment effects on mortality inform the shared-treatment effect parameter depends on the mortality rate within the study.

The functional component of the shared parameter model is a linear mixed effects model that adjusts for differences in ALSFRS-R slopes due to baseline covariates of time since onset, pre-baseline slope, edaravone and riluzole use. Like the treatment effect, these covariate effects are multiplicative effects on the slope. As such a value greater than

© 2025 Paganoni S et al. *JAMA Network Open*.

1 for a covariate indicates an increase in the rate of progression and a value less than one indicates a slowing in disease progression.

The primary analysis includes concurrent shared controls across regimens. It is expected that there will not be systematic differences in rates of progression in controls across regimens. The platform enrolls participants under the same master protocol, across the same sites, during the same time period, and with only very minor differences in regimen-specific inclusion/exclusion. However, minor differences across the shared controls may be present due to these minor differences in inclusion/exclusion and due to differences in the modes of administration. To account for these potential differences, the primary analysis is conducted within a Bayesian hierarchical meta-analytic framework. As such, the regimen-specific random effects on the rate of progression for controls are assumed to come from an underlying hierarchical distribution with unknown population mean and variability. The population mean and variability (hyper-parameters) within the hierarchical distribution are given a prior distribution, and posterior estimates are obtained from the data. If the variability in the rate of progression is estimated to be small, the model will borrow more information across regimens and the analysis will be similar to a pooled analysis across regimens. If the variability in the rate of progression is estimated to be large across regimens, the model will borrow less across regimens and the analysis will be similar to an independent analysis across regimens.
The Bayesian posterior distribution of all model parameters is calculated using Markov chain Monte Carlo (MCMC). We drew at least 500,000 samples from the posterior distribution with a thinning of 10. The exact number of samples from the MCMC was determined based on convergence diagnostics to ensure an adequate effective sample size of the posterior distribution.
Prespecified sensitivity analyses, including CAFS, which was employed as a sensitivity analysis, in addition to a secondary endpoint, were performed to assess the sensitivity of the results to the primary analysis population, modeling assumptions, and missing data assumptions as described in Statistical Analysis Plan (available as a Supplement).

Secondary analyses

SVC and HHD measures were assessed using both a repeated measures linear mixed model and random slopes model with the following covariates, centered to the analysis population: months since symptom onset, pre-baseline change in ALSFRS-R slope (delta-FRS), baseline riluzole and edaravone use, and their interaction with time. Models run on the full analysis set, which includes shared placebos from other regimens, random regimen-specific intercepts are included.

Each participant's vital status was verified at the time of the last patient last visit in the RCT. Rates of death, death-equivalent events (permanent assisted ventilation (PAV) defined as use of ventilation for >22 hours per day for more than 7 days in a row), and hospitalization were analyzed using a Cox proportional hazards model, with covariates of pre-baseline ALSFRS-R slope, months since symptom onset, riluzole and edaravone use at baseline, and age at baseline. A separate supportive analysis was run which included centered baseline serum NfL level and the interaction of centered baseline serum NfL level and visit as additional covariates.

Additional details are available in the Statistical Analysis Plan (available as a Supplement).

Estimand

The estimand of the primary analysis is the relative rate of disease progression (the "disease rate ratio" or DRR) of active treatment relative to placebo in the FAS population under an assumption that active treatment slows mean time to death or death equivalent by the same proportion as treatment slows the mean rate of functional progression as measured by change in ALSFRS-R total score over time. The estimand is defined by the following attributes:

- Treatment: zilucoplan administered subcutaneously once daily at a dosage of approximately 0.3 mg/kg vs. placebo.
- Population: FAS population whch includes all participants as they were randomized into this regimen, and shared controls from 3 other regimens as described in the methods section
- Variables: time to death or death equivalent and rate of change in ALSFRS-R total score from baseline to the Week 24 Visit.

© 2025 Paganoni S et al. *JAMA Network Open*.

- Intercurrent event 1: treatment discontinuation due to death: no ALSFRS-R data from participants who reach the death or death equivalent endpoint are included in the analysis, handled via mortality component in model, composite variable strategy approach.
- Intercurrent event 2: treatment discontinuation not due to death: handled via treatment policy approach, all data will be used including data collected during the placebo-controlled period after treatment discontinuation regardless of concomitant medication, for those participants who have not been censored due to mortality. Missing data post-treatment will not be imputed, handled via missing at random assumption.
- Population-level summary: mean ratio of hazard or progression rate of active treatment relative to placebo.

eReference

1- Zhao and Wang; Block urn design - a new randomization algorithm for sequential trials with two or more treatments and balanced or unbalanced allocation. Contemp Clin Trials. 2011 Nov;32(6):953-61

© 2025 Paganoni S et al. *JAMA Network Open*.
