# Supplementary material for: Efficacy and Safety of Zilucoplan in Amyotrophic Lateral Sclerosis: A Randomized Clinical Trial
Source: JAMA Netw Open. 2025 Feb 17;8(2):e2459058. doi: 10.1001/jamanetworkopen.2024.59058 (PMC11833520; doi:10.1001/jamanetworkopen.2024.59058)
Supplement: Supplement 3. — Nonauthor Collaborators. The HEALEY ALS Platform Trial Study Group Members [file jamanetwopen-e2459058-s003.pdf]

Supplemental Online Content: Nonauthor Collaborators
*First name, last name, and suffix (if applicable) are required and will appear in PubMed.

***Group Name(s): HEALEY ALS Platform Trial Study Group**

***First Name and Middle Initial(s)**	***Last Name**	***Suffix (eg, Jr, III)**	Academic Degrees	Institution	Location (city, state/province, country)	Role or Contribution, eg, chair, principal investigator	Group (if more than 1 Group listed in the byline) and/or Subgroup (eg, Steering Committee)
Douglas * deceased	Hayden		PhD	Massachusetts General Hospital, Harvard Medical School	Boston,MA, USA	Biostatistician	
Po-Ying	Lai		MS	Massachusetts General Hospital	Boston,MA, USA	Biostatistician	
Rachel A.	Donahue		MS	Massachusetts General Hospital	Boston,MA, USA	Biostatistician	
Hao-Wun	Chen		MS	Massachusetts General Hospital	Boston,MA, USA	Biostatistician	
Jianing	Wang		PhD	Massachusetts General Hospital; Harvard Medical School	Boston,MA, USA	Biostatistician	
Nithya	Mathai		DNP, APRN, FNP-C	Holy Cross Hospital	Fort Lauderdale, FL,USA	Sub-Investigator	
Gabriela	Lopes		NP	Holy Cross Hospital	Fort Lauderdale, FL,USA	Sub-Investigator	
Alexandra	McCaffrey		NP	Massachusetts General Hospital	Boston,MA, USA	Sub-Investigator	
Jennifer	Scalia		MSN, NP-C	Massachusetts General Hospital	Boston,MA, USA	Sub-Investigator	
Sarah	Luppino		MSN NP-BC	Massachusetts General Hospital	Boston,MA, USA	Sub-Investigator	
Clotilde	Lagier-Tourenne		MD, PhD	Massachusetts General Hospital	Boston,MA, USA	Scientific Advisory Board	
Ghazaleh	Sadri-Vakili		PhD	Massachusetts General Hospital	Boston,MA, USA	Scientific Advisory Board	
Stephen	Kolb		MD, PhD	Ohio State University	Columbus, OH,USA	Sub-Investigator	
Sarah	Heintzman		APRN-CNP, FNP-C, CCRC	Ohio State University	Columbus, OH,USA	Sub-Investigator	
Robert	Sufit		MD	Northwestern University	Evanston, IL, USA	Sub-Investigator	
April	Szymanski		APRN, ANP-BC	Northwestern University	Evanston, IL, USA	Sub-Investigator	
Liberty	Jenkins		MB ChB	California Pacific Medical Center	San Francisco, California,USA	Sub-Investigator	
Alan	Martin		MD	Texas Neurology, PA	Dallas, TX,USA	Sub-Investigator	
Ericka	(Simpson) Greene		MD	Houston Methodist Hospital	Houston, TX, USA	Sub-Investigator	
Jason R.	Thonhoff		MD, PhD	Houston Methodist Hospital	Houston, TX, USA	Sub-Investigator	
Bing	Liao		MD, MSc	Houston Methodist Hospital	Houston, TX, USA	Sub-Investigator	
Charles * deceased	Whitaker		MD	Hospital for Special Care	New Britain, CT,USA	Sub-Investigator	
Lora L.	Clawson		MSN, CRNP	Johns Hopkins University	Baltimore, MD, USA	Sub-Investigator	
Alpa	Uchil		MPH, MSN, CRNP	Johns Hopkins University	Baltimore, MD, USA	Sub-Investigator	
Kristen M.	Riley		PhD, CCRP	Johns Hopkins University	Baltimore, MD, USA	Sub-Investigator	
JinAe	Arneklev		CRNP	Johns Hopkins University	Baltimore, MD, USA	Sub-Investigator	

Supplemental Online Content: Nonauthor Collaborators
*First name, last name, and suffix (if applicable) are required and will appear in PubMed.

***First Name and Middle Initial(s)**	***Last Name**	***Suffix (eg, Jr, III)**	Academic Degrees	Institution	Location (city, state/province, country)	Role or Contribution, eg, chair, principal investigator	Group (if more than 1 Group listed in the byline) and/or Subgroup (eg, Steering Committee)
James	Grogan		MD	Penn State Milton S. Hershey Medical Center	Hershey, PA, USA	Sub-Investigator	
Xiaowei	Su		MD, PhD	Penn State Milton S. Hershey Medical Center	Hershey, PA, USA	Sub-Investigator	
Mansoureh	Mamarabadi		MD	Penn State Milton S. Hershey Medical Center	Hershey, PA, USA	Sub-Investigator	
Amber	Malcolm		NP	Washington University	St. Louis, MO, USA	Sub-Investigator	
Tracy	Bazan		MD	Providence ALS Clinic	Portland, OR, USA	Sub-Investigator	
Nassim	Rad		MD	University of Washington	Seattle, WA, USA	Sub-Investigator	
Leo H.	Wang		MD, PhD	University of Washington	Seattle, WA, USA	Sub-Investigator	
Eva L.	Feldman		MD, PhD	University of Michigan	Ann Arbor, MI, USA	Sub-Investigator	
Ezequiel	Piccione		MD	University of Nebraska Medical Center	Omaha,NE,USA	Sub-Investigator	
Pariwat	Thaisetthawatkul		MD	University of Nebraska Medical Center	Omaha,NE,USA	Sub-Investigator	
Constantine	Farmakidis		MD	University of Kansas Medical Center	Fairway,KS,USA	Sub-Investigator	
Duaa	Jabari		MD	University of Kansas Medical Center	Fairway,KS,USA	Sub-Investigator	
Jeffrey	Statland		MD	University of Kansas Medical Center	Fairway,KS,USA	Sub-Investigator	
Mamatha	Pasnoor		MD	University of Kansas Medical Center	Fairway,KS,USA	Sub-Investigator	
Mazen	Dimachkie		MD	University of Kansas Medical Center	Fairway,KS,USA	Sub-Investigator	
Robert H.	Brown, Jr.		MD, DPhil	University of Massachusetts, Worcester	Worecester,MA,USA	Sub-Investigator	
Mehdi	Ghasemi		MD, MPH	University of Massachusetts, Worcester	Worecester,MA,USA	Sub-Investigator	
Hajar	Houmani		DNP	University of Massachusetts, Worcester	Worecester,MA,USA	Sub-Investigator	
Catherine	Douthwright		PhD	University of Massachusetts, Worcester	Worecester,MA,USA	Sub-Investigator	
Kate	Daniello		MD	University of Massachusetts, Worcester	Worecester,MA,USA	Sub-Investigator	
Niraja	Suresh		MD	University of South Florida, College of Medicine	Tampa,FL,USA	Sub-Investigator	
Jerrica	Farias		MSN, APRN	University of South Florida, College of Medicine	Tampa,FL,USA	Sub-Investigator	
I-Hweii A.	Chen		MD PhD	University of South Florida, College of Medicine	Tampa,FL,USA	Sub-Investigator	
Piera	Pasinelli		PhD	Thomas Jefferson University	Philadelphia,PA,USA	Sub-Investigator	
Kara	Steijlen		MD	Henry Ford Health	Detroit,MI,USA	Sub-Investigator	
Ratna	Bhavaraju-Sanka		MD	UT Health San Antonio	San Antonio, TX,USA	Sub-Investigator	
Bill	Jacobsen		MD	Barrow Neurological Institute	Phoenix, AZ,USA	Sub-Investigator	
Jourdan	Milliard		NP	Barrow Neurological Institute	Phoenix, AZ,USA	Sub-Investigator	
Robert	Bowser		PhD	Barrow Neurological Institute	Phoenix, AZ,USA	Sub-Investigator	
Anahita	Deboo		MD	Temple University	Philadelphia,PA,USA	Sub-Investigator	

Supplemental Online Content: Nonauthor Collaborators
*First name, last name, and suffix (if applicable) are required and will appear in PubMed.

***First Name and Middle Initial(s)**	***Last Name**	***Suffix (eg, Jr, III)**	Academic Degrees	Institution	Location (city, state/province, country)	Role or Contribution, eg, chair, principal investigator	Group (if more than 1 Group listed in the byline) and/or Subgroup (eg, Steering Committee)
Michael S.	Cartwright		MD, MS	Wake Forest School of Medicine	Winston-Salem, NC,USA	Sub-Investigator	
Christopher	Nance		MD	University of Iowa	Iowa City, IA,USA	Sub-Investigator	
Ludwig	Gutmann		MD	University of Iowa	Iowa City, IA,USA	Sub-Investigator	
Julia	Yasek		NP	Columbia University Medical Center	New York, NY,USA	Sub-Investigator	
Matthew	Harms		MD	Columbia University Medical Center	New York, NY,USA	Sub-Investigator	
Matthew	Burford		MD	Cedars-Sinai Medical Center	Los Angeles, CA,USA	Sub-Investigator	
Frank	Diaz		MD, PhD	Cedars-Sinai Medical Center	Los Angeles, CA,USA	Sub-Investigator	
David	Shrilla		DO	Medical College of Wisconsin	Milwaukee, WI,USA	Sub-Investigator	
Goran	Rakocevic		MD	University of Virginia	Charlottesville, VA,USA	Sub-Investigator	
Sarah	Jones		MD	University of Virginia	Charlottesville, VA,USA	Sub-Investigator	
Guillermo	Solorzano		MD	University of Virginia	Charlottesville, VA,USA	Sub-Investigator	
Xiaoyan	Li		MD, PhD	Duke University	Durham, NC, USA	Sub-Investigator	
Zabeen	Mahuwala		MD	University of Kentucky	Lexington, KY, USA	Sub-Investigator	
Vishakhadatta (Vish) Mathur	Kumaraswamy		MD	University of Kentucky	Lexington, KY, USA	Sub-Investigator	
Colin	Quinn		MD	University of Pennsylvania	Philadelphia,PA,USA	Principal Investigator	
Michael	Baer		MD	University of Pennsylvania	Philadelphia,PA,USA	Sub-Investigator	
David	Borg		MD	Loma Linda University School of Medicine	Loma Linda, CA, USA	Sub-Investigator	
Karthikeyan	Bhuvaneswaran		DO	Loma Linda University School of Medicine	Loma Linda, CA, USA	Sub-Investigator	
Jasdeep	Kaur		FNP RN	Loma Linda University School of Medicine	Loma Linda, CA, USA	Sub-Investigator	
Sam	Maiser		MD	University of Minnesota/Twin Cities ALS Research Consortium	Minneapolis, MN, USA	Sub-Investigator	
Seward B.	Rutkove		MD	Beth Israel Deaconess Medical Center	Boston,MA, USA	Principal Investigator	
Andrew	Mundwiler		MD	Spectrum Health Medical Group	Grand Rapids,MI,USA	Sub-Investigator	
Jenny A.	Meyer		MD	SUNY Upstate	Syracuse, NY, USA	Sub-Investigator	
Pooja	Rao		MD	Ochsner Health System	New Orleans,LA,USA	Sub-Investigator	
Betty	Soliven		MD	University of Chicago	Chicago,IL,USA	Sub-Investigator	
Raymond	Roos		MD	University of Chicago	Chicago,IL,USA	Sub-Investigator	
Ali A.	Habib		MD	University of California, Irvine Medical Center	Orange,CA,USA	Sub-Investigator	

Supplemental Online Content: Nonauthor Collaborators
*First name, last name, and suffix (if applicable) are required and will appear in PubMed.

***First Name and Middle Initial(s)**	***Last Name**	***Suffix (eg, Jr, III)**	Academic Degrees	Institution	Location (city, state/province, country)	Role or Contribution, eg, chair, principal investigator	Group (if more than 1 Group listed in the byline) and/or Subgroup (eg, Steering Committee)
Tahseen	Mozaffar		MD	University of California, Irvine Medical Center	Orange,CA,USA	Sub-Investigator	
Manisha Kak	Korb		MD	University of California, Irvine Medical Center	Orange,CA,USA	Sub-Investigator	
Jeffrey	Mullen		MD	University of California, Irvine Medical Center	Orange,CA,USA	Sub-Investigator	
Elijah	Stommel		MD	Dartmouth-Hitchcock Medical Center	Lebanon,NH,USA	Sub-Investigator	
Nathaniel M	Robbins		MD	Dartmouth-Hitchcock Medical Center	Lebanon,NH,USA	Sub-Investigator	
Nathan	Carberry		MD	University of Miami	Miami,FL,USA	Sub-Investigator	
Volkan	Granit		MD	University of Miami (currently at Biohaven Pharmaceuticals)	Miami,FL,USA	Sub-Investigator	
Raghav	Govindarajan		MD	University of Missouri	Columbia,MO,USA	Principal Investigator	
Bjorn	Oskarsson		MD	Mayo Clinic - Jacksonville	Jacksonville,FL,USA	Sub-Investigator	
Leila	Darki		MD	University of Southern California	Los Angeles, CA,USA	Sub-Investigator	
Rodrigo	Rodriguez		MD	University of Southern California	Los Angeles, CA,USA	Sub-Investigator	
Miguel	Chuquilin		MD	University of Florida Gainesville	Gainesville, FL, USA	Sub-Investigator	
Whitney	McNeely		APRN	University of Florida Gainesville	Gainesville, FL, USA	Sub-Investigator	
Montserrat	Diaz-Abad		MD	University of Maryland School of Medicine	Baltimore, MD, USA	Sub-Investigator	
Peter H.	Jin		MD	University of Maryland School of Medicine	Baltimore, MD, USA	Sub-Investigator	
Chandana	Chauhan		MD	University of Maryland School of Medicine	Baltimore, MD, USA	Sub-Investigator	
James	Bobenhouse		MD	Neurology Associates	Lincoln,NE,USA	Sub-Investigator	
Nathan P.	Staff		MD, PhD	Mayo Clinic - Rochester, MN	Rochester,MN,USA	Sub-Investigator	
Ghazala	Hayat		MD	Saint Louis University	St. Louis, MO, USA	Principal Investigator	
Luisa	Arroyave			Massachusetts General Hospital	Boston,MA, USA	Project Management	
Abbey	Bailey		BA	Massachusetts General Hospital	Boston,MA, USA	Project Management	
Jesse	Bailey		BA	Massachusetts General Hospital	Boston,MA, USA	Project Management	
Victoria	Barlow		MS	Massachusetts General Hospital	Boston,MA, USA	Project Management	

Supplemental Online Content: Nonauthor Collaborators
*First name, last name, and suffix (if applicable) are required and will appear in PubMed.

***First Name and Middle Initial(s)**	***Last Name**	***Suffix (eg, Jr, III)**	Academic Degrees	Institution	Location (city, state/province, country)	Role or Contribution, eg, chair, principal investigator	Group (if more than 1 Group listed in the byline) and/or Subgroup (eg, Steering Committee)
Allison	Bulat			Massachusetts General Hospital	Boston,MA, USA	Patient Navigator	
Genevive	Changkuon		MHS	Massachusetts General Hospital	Boston,MA, USA	Data Management	
Melissa	Cirino			Massachusetts General Hospital	Boston,MA, USA	sIRB	
Cristina	Deignan			Massachusetts General Hospital	Boston,MA, USA	System Management	
Emma	Deirmendjian		RPh	Massachusetts General Hospital	Boston,MA, USA	Quality Assurance	
Annette	De Mattos		MPH	Massachusetts General Hospital	Boston,MA, USA	Grants/Contract Management	
Sofia	DiStefano		MS	Massachusetts General Hospital	Boston,MA, USA	Project Management	
Kristin	Drake		MS, MBA	Massachusetts General Hospital	Boston,MA, USA	Contract Management	
Michaela	Estes		MPH	Massachusetts General Hospital	Boston,MA, USA	Data Management	
Kenneth	Faulconer			Massachusetts General Hospital	Boston,MA, USA	System Management	
Precious	Figueroa-Szostek			Massachusetts General Hospital	Boston,MA, USA	Grants Management	
Tessa	Garozzo			Massachusetts General Hospital	Boston,MA, USA	Quality Assurance	
Meredith Gibbons	Hasenoehrl		PhD	Massachusetts General Hospital	Boston,MA, USA	Grants Management	
Jennifer	Henrique			Massachusetts General Hospital	Boston,MA, USA	Project Management	
Natalie	Henrique			Massachusetts General Hospital	Boston,MA, USA	sIRB	
Samuel	Hurwitz			Massachusetts General Hospital	Boston,MA, USA	Data Management	
Courtney	Igne		MS	Massachusetts General Hospital	Boston,MA, USA	Project Management	
Liam	Irwin			Massachusetts General Hospital	Boston,MA, USA	System Management	
Katie	Jentoft			Massachusetts General Hospital	Boston,MA, USA	Data Management	
Boglarka	Jordan		MPH	Massachusetts General Hospital	Boston,MA, USA	Project Management	
Igor	Katsovskiy		MS	Massachusetts General Hospital	Boston,MA, USA	System Management	
Olga	Kharakozova			Massachusetts General Hospital	Boston,MA, USA	System Management	
Taylor	Kolvek			Massachusetts General Hospital	Boston,MA, USA	Project Management	

Supplemental Online Content: Nonauthor Collaborators
*First name, last name, and suffix (if applicable) are required and will appear in PubMed.

*First Name and Middle Initial(s)	*Last Name	*Suffix (eg, Jr, III)	Academic Degrees	Institution	Location (city, state/province, country)	Role or Contribution, eg, chair, principal investigator	Group (if more than 1 Group listed in the byline) and/or Subgroup (eg, Steering Committee)
Alexander	Korin			Massachusetts General Hospital	Boston,MA, USA	System Management	
Thuong	La			Massachusetts General Hospital	Boston,MA, USA	Data Management	
Haining	Li			Massachusetts General Hospital	Boston,MA, USA	Data Management	
Joey	Nguyen		M.Ed.	Massachusetts General Hospital	Boston,MA, USA	Grants Management	
Ilya	Novak			Massachusetts General Hospital	Boston,MA, USA	System Management	
Ricardo	Ortiz		MBA	Massachusetts General Hospital	Boston,MA, USA	Grants Management	
Joe	Ostrow			Massachusetts General Hospital	Boston,MA, USA	Data Management	
Jaclyn	Pagliaro		MPH	Massachusetts General Hospital	Boston,MA, USA	Project Management	
Jack	Palillo		MPH	Massachusetts General Hospital	Boston,MA, USA	Data Management	
Payal	Patel			Massachusetts General Hospital	Boston,MA, USA	System Management	
Janae	Patterson		MS	Massachusetts General Hospital	Boston,MA, USA	Grants/Contract Management	
Minh	Phan			Massachusetts General Hospital	Boston,MA, USA	Data Management	
Najla	Popel			Massachusetts General Hospital	Boston,MA, USA	Data Management	
Serena	Proueng			Massachusetts General Hospital	Boston,MA, USA	Project Management	
Jesse	Rosenthal			Massachusetts General Hospital	Boston,MA, USA	Project Management	
Catherine	Small			Massachusetts General Hospital	Boston,MA, USA	Patient Navigator	
Natalia	Tarasenko			Massachusetts General Hospital	Boston,MA, USA	System Management	
Mirna	Thomas		MBA	Massachusetts General Hospital	Boston,MA, USA	Data Management	
Prasha	Vigneswaran		MS	Massachusetts General Hospital	Boston,MA, USA	System Management	
Yusra	Wahab			Massachusetts General Hospital	Boston,MA, USA	System Management	
Isaac	Whitworth			Massachusetts General Hospital	Boston,MA, USA	System Management	
Spencer	Wright			Massachusetts General Hospital	Boston,MA, USA	Project Management	
Mariah	Connolly		BS, CCRA	Barrow Neurological Institute	Phoenix, AZ,USA	Site Monitoring	
Diana	De Santiago		MHA	Barrow Neurological Institute	Phoenix, AZ,USA	Site Monitoring	

Supplemental Online Content: Nonauthor Collaborators
*First name, last name, and suffix (if applicable) are required and will appear in PubMed.

***First Name and Middle Initial(s)**	***Last Name**	***Suffix (eg, Jr, III)**	Academic Degrees	Institution	Location (city, state/province, country)	Role or Contribution, eg, chair, principal investigator	Group (if more than 1 Group listed in the byline) and/or Subgroup (eg, Steering Committee)
Adrian	Felix		MSc, MD	Barrow Neurological Institute	Phoenix, AZ,USA	Site Monitoring	
Karly	Garrett		BS	Barrow Neurological Institute	Phoenix, AZ,USA	Site Monitoring	
Jenny	Hamilton		BA, CCRC	Barrow Neurological Institute	Phoenix, AZ,USA	Site Monitoring	
Kamran	Khan		BS	Barrow Neurological Institute	Phoenix, AZ,USA	Site Monitoring	
Marlee	Lovett		BS	Barrow Neurological Institute	Phoenix, AZ,USA	Site Monitoring	
Linda	Nelson		MPH	Barrow Neurological Institute	Phoenix, AZ,USA	Site Monitoring	
Marissa	Pabon		BA	Barrow Neurological Institute	Phoenix, AZ,USA	Site Monitoring	
Diana	Rede		BS	Barrow Neurological Institute	Phoenix, AZ,USA	Site Monitoring	
Patrick	Bolger		R.Ph., M.B.A.	Clinical Materials Services Unit (University of Rochester)	Rochester, NY,USA	Central Pharmacy	
Ahmed	Fetouh		PharmD, MBA	Clinical Materials Services Unit (University of Rochester)	Rochester, NY,USA	Central Pharmacy	
Joan	Woodcook		BS	Clinical Materials Services Unit (University of Rochester)	Rochester, NY,USA	Central Pharmacy	
Cornelia	Kamp		MBA	Clinical Materials Services Unit (University of Rochester) and Clintrex Research Corporation	Rochester, NY,USA	Central Pharmacy // DSMB	
Julie	Kennedy		RN, CCRP	Clintrex Research Corporation	Sarasota, FL,USA	DSMB	
Andrew	McGarry		MD	Clintrex Research Corporation	Sarasota, FL,USA	Medical Monitoring	
Margherita	Torti		MD	Clintrex Research Corporation	Sarasota, FL,USA	Medical Monitoring	
