# Supplementary material for: Efficacy and Safety of Zilucoplan in Amyotrophic Lateral Sclerosis: A Randomized Clinical Trial
Source: JAMA Netw Open. 2025 Feb 17;8(2):e2459058. doi: 10.1001/jamanetworkopen.2024.59058 (PMC11833520; doi:10.1001/jamanetworkopen.2024.59058)
Supplement: Supplement 4. — Data Sharing Statement [file jamanetwopen-e2459058-s004.pdf]

Data Sharing Statement

Paganoni. Efficacy and Safety of Zilucoplan in Amyotrophic Lateral Sclerosis. *JAMA Netw Open*. Published February 17, 2025. doi:10.1001/jamanetworkopen.2024.59058

Data
Additional Information: Clinical Trial Identifier NCT04297683
Data available: No

Additional Information
Explanation for why data not available: Data Sharing Statement All data and report requests should be submitted to the HEALEY ALS Platform Trial Data and Report Sharing Review Committee at HealeyAMGCenterforALS@mgh.harvard.edu There will be a period of time where the Healey Placebo Data will not be shared outside of the Sponsor, Regimen Partners, Writing Group Leadership, and regulators as outlined in the HEALEY ALS Platform Trial Data and Report Sharing Policy (add link) to protect the scientific integrity of the active regimens in the HEALEY ALS Platform Trial. Statistical analyses and design simulations were programmed in SAS v9.4 or later, R v4.0 or later, or JAGS v4.3 or later; code is not publicly available at the present time as the trial is still ongoing.
